# A Jump-from-Cavity Pyrophosphate Ion Release Assisted by a Key Lysine Residue in T7 RNA Polymerase Transcription Elongation

**DOI:** 10.1371/journal.pcbi.1004624

**Published:** 2015-11-24

**Authors:** Lin-Tai Da, Chao E, Baogen Duan, Chuanbiao Zhang, Xin Zhou, Jin Yu

**Affiliations:** 1 Department of Physics and Institute of Molecular Biophysics, Florida State University, Tallahassee, Florida, United States of America; 2 Beijing Computational Science Research Center, Beijing, China; 3 School of Physics, University of the Chinese Academy of Sciences, Beijing, China; UNC Charlotte, UNITED STATES

## Abstract

Pyrophosphate ion (PPi) release during transcription elongation is a signature step in each nucleotide addition cycle. The kinetics and energetics of the process as well as how it proceeds with substantial conformational changes of the polymerase complex determine the mechano-chemical coupling mechanism of the transcription elongation. Here we investigated detailed dynamics of the PPi release process in a single-subunit RNA polymerase (RNAP) from bacteriophage T7, implementing all-atom molecular dynamics (MD) simulations. We obtained a jump-from-cavity kinetic model of the PPi release utilizing extensive nanosecond MD simulations. We found that the PPi release in T7 RNAP is initiated by the PPi dissociation from two catalytic aspartic acids, followed by a comparatively slow jump-from-cavity activation process. Combining with a number of microsecond long MD simulations, we also found that the activation process is hindered by charged residue associations as well as by local steric and hydrogen bond interactions. On the other hand, the activation is greatly assisted by a highly flexible lysine residue Lys472 that swings its side chain to pull PPi out. The mechanism can apply in general to single subunit RNA and DNA polymerases with similar molecular structures and conserved key residues. Remarkably, the flexible lysine or arginine residue appears to be a universal module that assists the PPi release even in multi-subunit RNAPs with charge facilitated hopping mechanisms. We also noticed that the PPi release is not tightly coupled to opening motions of an O-helix on the fingers domain of T7 RNAP according to the microsecond MD simulations. Our study thus supports the Brownian ratchet scenario of the mechano-chemical coupling in the transcription elongation of the single-subunit polymerase.

## Author Summary

RNA polymerases (RNAPs) are usually recognized to work as Brownian ratchet machines, with mechanical movements and chemical reactions loosely coupled during transcription elongation. Nevertheless, structural studies on the single-subunit T7 RNAP had suggested an alternative power stroke scenario, which requires the PPi product release reaction tightly couples with or drives the polymerase translocation. To resolve the conflicting views in the mechano-chemical coupling scenario, we conducted atomistic molecular dynamics (MD) simulations to investigate the PPi release mechanism of T7 RNAP. Using a large number of nanosecond short simulations, we constructed the Markov state model (MSM) and found that the PPi release undergoes a jump-from-cavity activation process, unlikely to further support the translocation. The MSM implementations to multi-subunit RNAPs previously demonstrated instead the charge facilitated hopping mechanisms of the PPi release. To further explore essential slow motions during the PPi release, we also performed microsecond long MD simulations. The PPi release does not appear to be tightly coupled to the rotational opening of an O-helix that is directly tied to the polymerase translocation. Hence, the study again disfavors the power stroke scenario. Remarkably, we discovered a key residue Lys472 that is able to assist PPi to jump out of the cavity. The mechanism can be general to a group of polymerases sharing conserved structural features with T7 RNAP, while the lysine or arginine module to assist the PPi release can be universal to both the single and multi-subunit RNAPs, even though the overall structural features and the PPi release mechanisms are significantly different.

## Introduction

Transcription elongation is a continuous process of producing messenger RNA as an RNA polymerase (RNAP) moves along double stranded (ds) DNA, copying information from the DNA template to synthesize an RNA strand [1–3]. For each nucleotide addition cycle (NAC) of the transcription elongation, a nucleoside triphosphate (NTP) is recruited into the active site of RNAP. Then phosphoryl transfer reaction happens which leads to the addition of the nucleoside monophosphate (NMP) to the existing RNA strand, followed by the release of a pyrophosphate ion (PPi). The PPi release hence serves as a nice signal for detecting each NAC, for example, in real-time DNA sequencing [4,5].

Although the PPi release in RNAP can be detected, how it happens in structural dynamics detail remains elusive from existing experimental measurements. Since the polymerase works as a molecular motor driven by chemical free energy from each NAC, how the PPi release after catalysis couples with essential conformation changes and translocation of the polymerase determines the mechano-chemical coupling nature of the motor system [1,6–9]. In studying the RNAP structures from bacteriophage T7, it was suggested that the PPi release directly drives the polymerase translocation [7]. Such a mechanism is called the ‘power stroke’ (PS), as the product release reaction couples tightly to the mechanical movement of the protein [10,11]. On the other hand, single molecule measurements implementing optical-tweezer forces on RNAPs, for single or multi-subunit ones, consistently suggested a ‘Brownian ratchet’ (BR) mechanism [12–14], in which the translocation proceeds in Brownian motions, thermally activated without being energetically coupled to the preceding PPi release. As such, it is necessary to clarify whether T7 RNAP makes an exception to the BR type RNAP motors. Besides, studies that are able to zoom into structural detail and to characterize kinetic or energetics of the process are highly demanded for understanding the basics of the transcription engine.

T7 RNAP is a prototypical single subunit polymerase that works self-sufficiently without transcription factors. It maintains high promoter specificity and drives transcription strongly [15]. T7 RNAP has been widely utilized in protein over-expression systems as well as in synthetic gene circuits [16,17]. It is also characterized well from structural and kinetic studies at single molecule level [7,14,18–22] as well as from biochemical ensemble measurements [6,23,24]. The PS mechanism was suggested based on the high-resolution structural studies [7]. It is observed that a flexible fingers domain with five alpha helices including an O-helix adopts two major conformations, the closed and open forms in each NAC. In the PS mechanism, a transition from the closed to open form was supposed to couple the PPi release to the polymerase translocation with a significant free energy decrease. Nevertheless, in the single molecule force measurements of T7 RNAP, the elongation rates measured at variable NTP concentrations and forces were fitted to a kinetic scheme, yielding only a very small free energy bias upon the translocation (~ 1k_B_T) [14,20], supporting the BR rather than the PS scenario. Furthermore, transient state kinetics measurements on the T7 RNAP transcription have also been conducted [23]. The study suggested that an isomerization from the open to the closed form prior to the catalysis is rate limiting, while the steps afterwards including the PPi release and translocation are fast. The study accordingly provides a kinetic framework to investigate the structure-function detail of the transcription elongation in T7 RNAP. The dissociation constant of PPi was determined at ~ 1.2 mM [23].

Recently, computational work combining extensive all-atom molecular dynamics (MD) simulations with the Markov state model (MSM) has been conducted to study the PPi release in multi-subunit RNAPs from bacteria to yeast [25,26]. The advantage of implementing the MSM is to efficiently group high-dimensional data and extract kinetic information from a large number of short MD simulations sampled widely in the conformational space [27–31]. One big challenge the conventional MD simulations face with is to reach physiologically relevant timescales, especially for the system as large as the RNAPs (system size of a hundred thousand to a million atoms, solvated in explicit water). The MSM had been implemented successfully to describe relatively long dynamics in protein [32,33] and RNA folding [34], receptor-ligand binding [35–37], etc.. The MSM constructed for the multi-subunit RNAPs suggested that the PPi release happens at around a microsecond [25,26], faster than the polymerase translocation (estimated to be tens of microseconds at least) [38]. In the multi-subunit RNAPs, there is an important structure called trigger loop (TL) [39,40] that folds (closes) and unfolds (opens) upon the NTP binding and PPi release, respectively, analogous to the O-helix closing and opening in the single-subunit T7 RNAP. In the yeast RNAPII, it was noticed that the TL increases its fluctuation after catalysis to aid the PPi release, which occurs through a hopping mode whereby four meta-stable states were identified [26]. In comparison, a relatively simple two-state model of the PPi release was discovered in bacterial RNAP [25]. The TL only slightly opens in the yeast RNAP II upon the PPi release, while the TL does not open in a same way in the bacterial RNAP [25,26].

Computational studies on single-subunit polymerases have also been conducted recently [41–47]. The structurally similar single-subunit polymerases include not only RNAPs from bacteriophage and human mitochondria, but also a group of DNA polymerases (DNAPs). The MD study on the translocation mechanism of DNAP I [44] showed that the PPi release facilitates the opening transition of the fingers domain. The authors therefore suggested that the PPi release triggers the translocation by facilitating the domain opening transition of the polymerase [44]. More recently, structured-based kinetic modeling and all-atom MD simulations on T7 RNAP have been conducted [42,43]. The kinetic modeling was built upon single molecule force measurements on the T7 RNAP transcription elongation[14,20], supporting the BR mechanism [43]. The ensued MD study focused on nucleotide selection prior to the catalysis for transcription fidelity control [42]. Further computational studies on the post-catalysis coordination from the PPi release to the translocation are highly desired, therefore, for a complete description of the elongation cycle and for resolving the conflicting views on the mechano-chemical coupling of the transcription machine.

**Fig 1:**
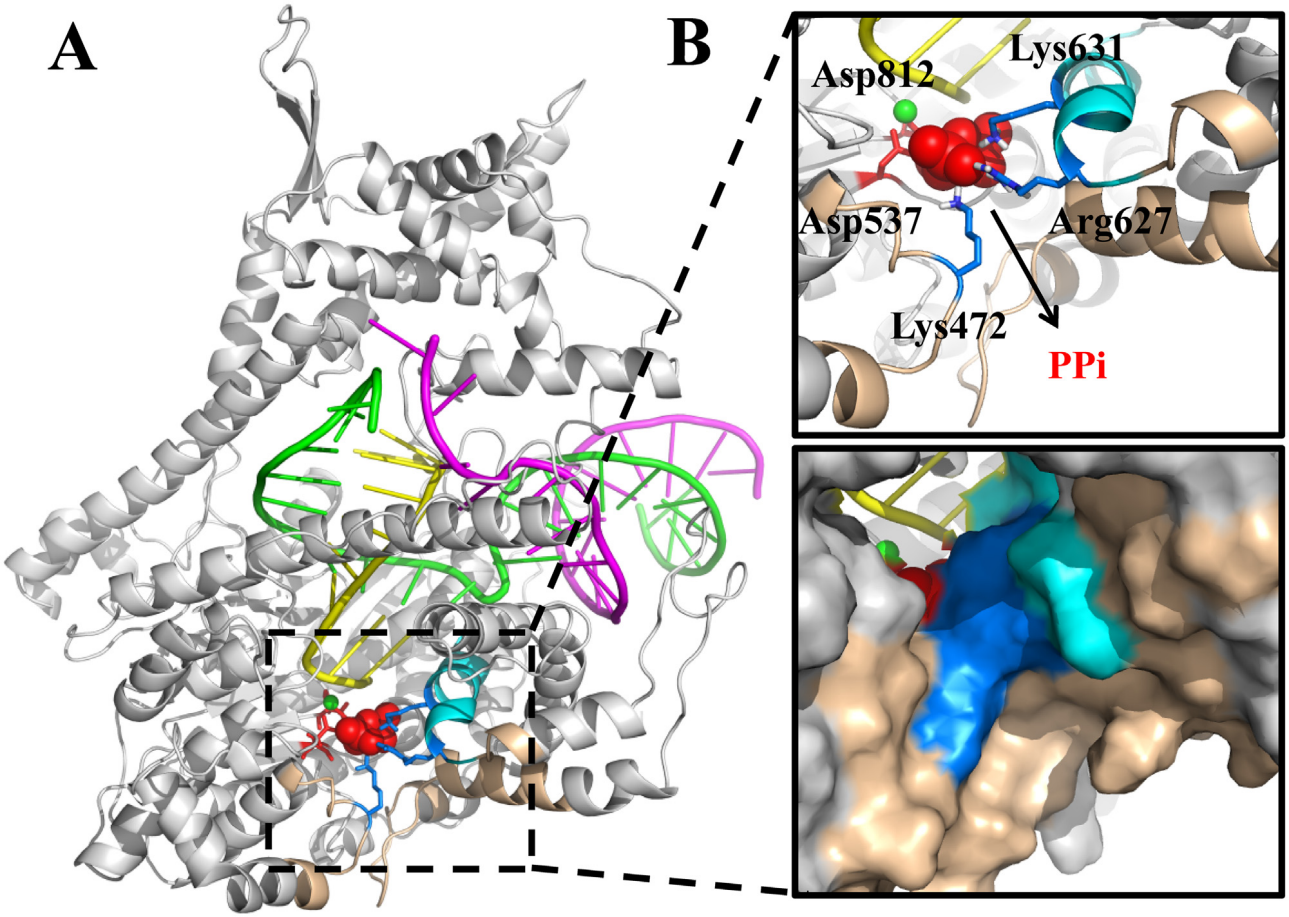
The modeled PPi-bound T7 RNA elongation complex. (A) The overall structure of the complex (protein in white; DNA non-template strand in purple; DNA template strand in green; RNA strand in yellow). (B) The zoomed in view of the active site, with essential amino acids shown in the upper panel (blue licorice: positively charged; red licorice: negatively charged). PPi is shown in red spheres. The Mg^2+^ in the active site is in green sphere. The O-helix is shown in cyan and the PPi release channel is highlighted in wheat color. The lower panel provides a surface representation.

In the single-subunit T7 RNAP, the active site is buried inside the polymerase within a distance of ~15Å away from the bulk water. Right after the catalysis, the negatively charged PPi sits inside a cavity, surrounded by three positively charged residues (Lys631, Arg627, and Lys472) from the outside, as well as by two negatively charged residue (Asp537 and Asp812; through a magnesium ion bound with PPi) from the inside (see Fig 1). Accordingly, the releasing pathway of the PPi group from the active site is blocked by the side chains of the above residues. The structural features of the PPi release channel appear quite different from that of the multi-subunit RNAPs, which have a fairly long pore region with a length of ~30 Å. A comparative study of the PPi release from the viral T7 RNAP with that from the bacteria and high organisms would then also help to elucidate how variable structural designs accommodate a convergent bio-molecular function, achieved through enzyme evolution from different species.

In this work, we started from an atomistic structure of the T7 RNAP elongation complex in the PPi-bound product state and conducted extensive MD simulations. The MSM was then constructed to extract kinetic information from the grouped snapshots sampled along the PPi release pathways. Comparing to the four-state and two-state PPi hopping model proposed previously for the multi-subunit RNAPs, we investigated the PPi release mechanism in T7 RNAP and built a corresponding model, concerning (1) how many metabstable states exist during the PPi release, (2) is the energetics going up or down along the release path, and (3) what is the rate-limiting transition in the full process. We obtained a jump-from-cavity kinetic model of the PPi release from the extensive simulations, with two relatively stable states inside the cavity, and one marginally stable state at the exit. We also found that the PPi jump from the cavity is rate limiting over a microsecond. In addition, we performed microsecond long MD simulations in both unperturbed and modified conditions. With that we identified which slow motions essentially couple with or impact on the PPi release. Overall, we demonstrated that the PPi release in T7 RNAP is a jump-from-cavity activation process closely assisted by a flexible lysine residue, without being tightly coupled to the opening of the O-helix to further support the translocation. In the end, we compare T7 RNAP with other RNA or DNA polymerases on conserved structural features that are key to the PPi release.

## Results

From a hundred of short MD simulations (aggregated to ~2 microsecond), we built a kinetic model of the PPi release by constructing the MSM. Next, essential slow motions around the active site, including both the O-helix opening and Lys472 side chain swing, were investigated in microsecond long MD simulations, in an unperturbed PPi-bound RNAP complex and two modified controlled systems, by either turning off charges of several key residues or removing the PPi group from the active site.

### The MSM reveals a jump-from-cavity mechanism of the PPi release

To fully sample the PPi release process, we first conducted steered MD (SMD) simulations along five potential pathways to pull the PPi group from inside around the active site toward the outside into the bulk water. To eliminate the bias introduced from the SMD simulation, the conformations sampled along the pulling pathways were grouped into tens of clusters. From each cluster, we then performed nanosecond MD simulations starting from centered conformations (see Methods). A total of ~100 MD trajectories were collected with a sum of ~1,000,000 snapshots aligned, so that the RMSDs of the PPi group were calculated to track the PPi location. Alternatively (see **Supporting Information** and S1A Fig), one can also measure the distance between the PPi group and a Mg^2+^ ion (MgA) kept inside the active site.

**Fig 2:**
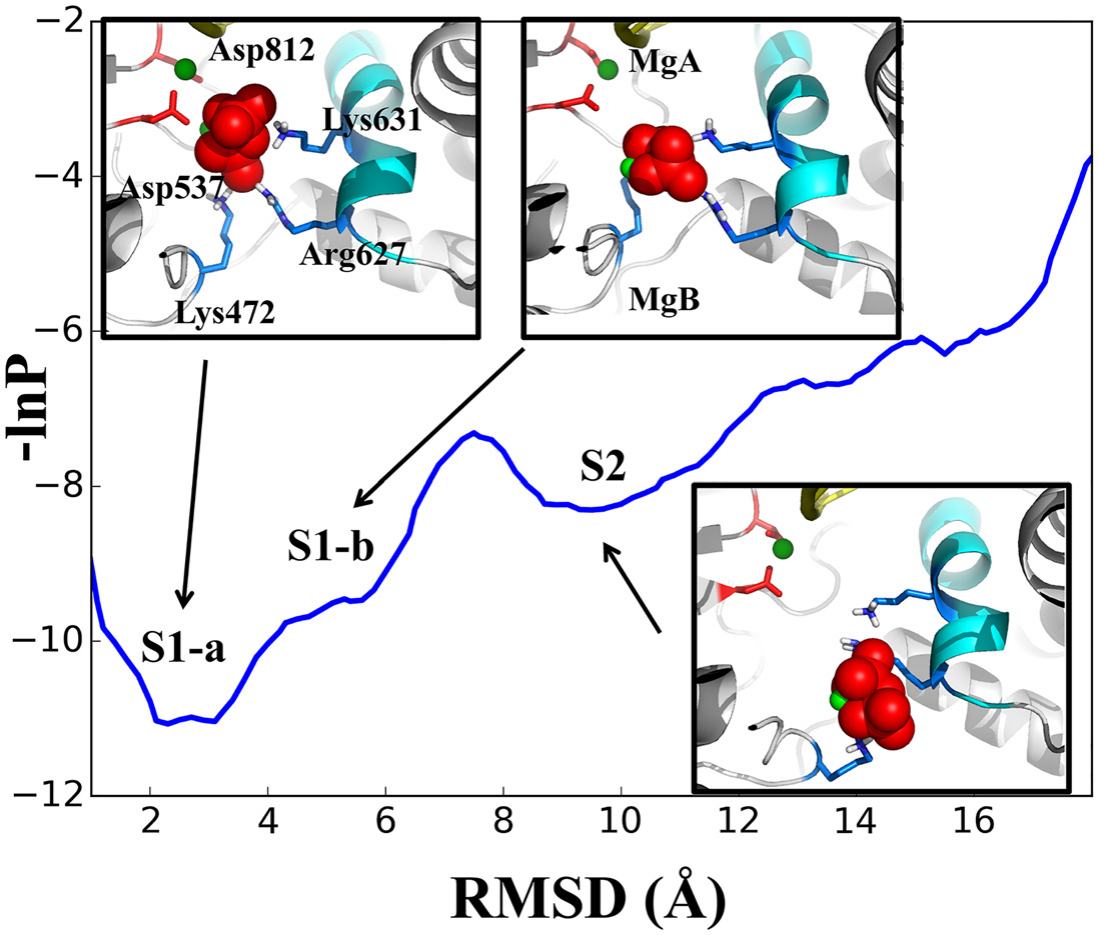
The population density profile for conformations sampled along the simulated PPi release pathways. The profile is shown on a potential-like surface along the PPi RMSD coordinate. Three population states are identified: **S1a**, **S1b**, and **S2**, with representative snapshots shown for each state. The color scheme in the structure is the same as that in Fig 1. The convergence tests of the profile is provided in SI S2 Fig.

We firstly mapped the sampled population density along the PPi RMSD coordinate. As shown in Fig 2, one can identify three population states on the profile, centered at ~ 3 Å, 6 Å, and 10 Å along the RMSD coordinate, and labeled as **S1a**, **S1b**, and **S2**, respectively. For each state, we provide one representative snapshot. In **S1a**, the PPi group is grabbed by three positively charged residues: Lys631, Arg627, and Lys472, as well as by two negatively charged residues: Asp537 and Asp812, through a Mg^2+^ ion (MgB) bound with PPi. These two aspartic acids are highly conserved in RNA polymerases, and play essential roles in catalysis [48,49]. Separation of PPi from these two aspartic acids is necessary for the PPi release, and this separation becomes visible in **S1b**. We also projected the sampled conformations along the PPi RMSD and the distance between the PPi-bound MgB and one oxygen of Asp537 (see S1B Fig). While in **S2**, the PPi group is not only separated far from Asp537 and Asp812, but also loses the contact with Lys631. In addition, the PPi group in **S2** forms marginally stable interactions with Lys472 and Arg627, which locate at the exit to the bulk water (see Fig 2). We also conducted convergence tests for the sampled population distributions (see S2 Fig).

**Fig 3:**
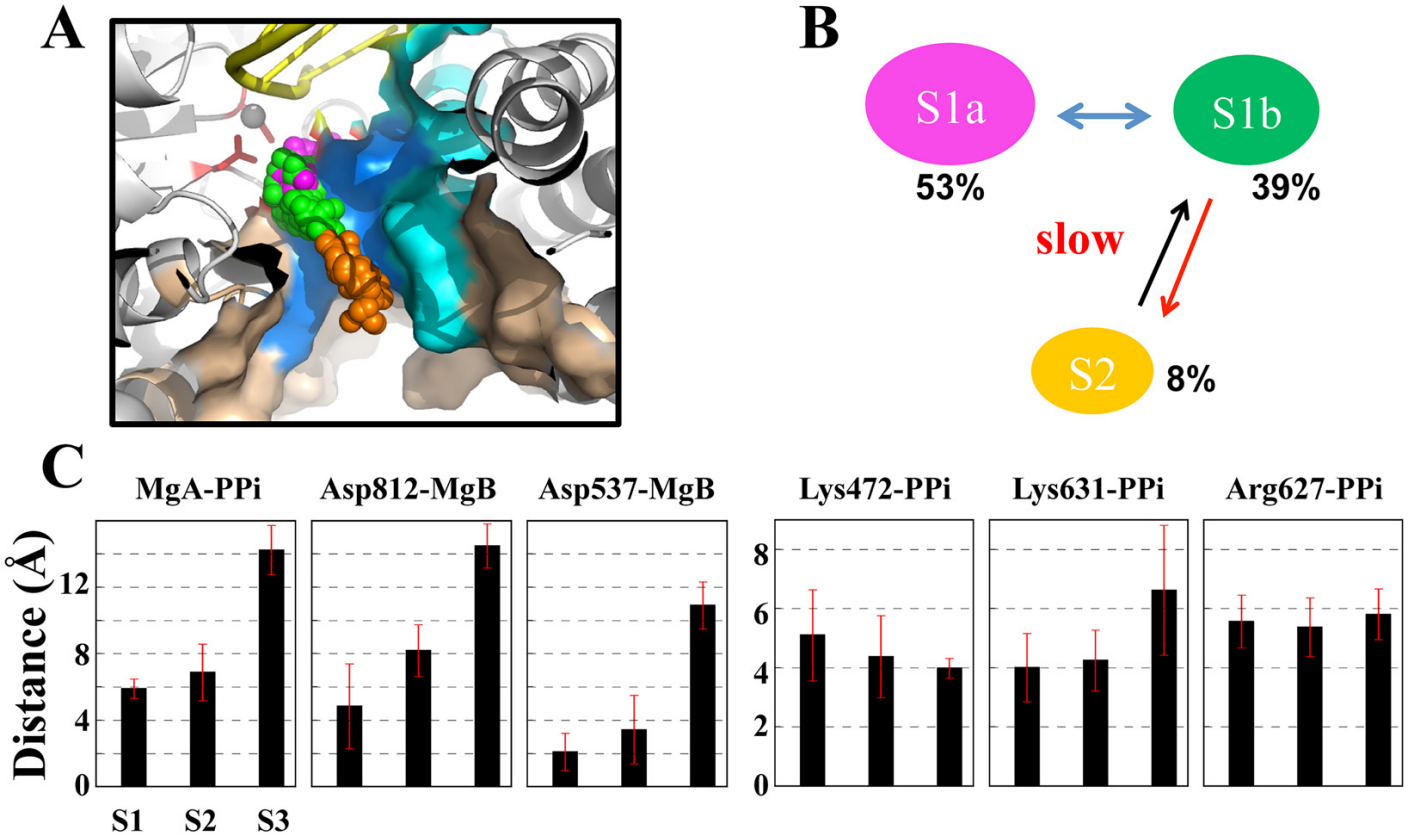
The MSM construction for the PPi release in the T7 RNAP elongation. (A) A structural view of the PPi distribution (shown in spheres) sampled from the MD simulations. Each sphere represents the center of the mass of the PPi group. The distribution was generated from randomly chosen MD snapshots for states **S1a** (in purple sphere), **S1b** (in green), and **S2** (in orange). (B) The three-state macrostate representation generated from the MSM construction. The equilibrium populations from the MSM are indicated. (C) Distance analyses between several key residues and the PPi group in the three-state representation.

We next constructed the MSM based on all the conformations sampled from the short MD simulations by firstly clustering these conformations into hundreds of microstates. We then further lumped these microstates into three macrostates in order to visualize key metastable states (see Methods and Fig 3A and 3B). The three states match well with the above mapping onto **S1a** (~53%), **S1b** (~39%), and **S2** (~8%). Hence, one can see that the total ‘inside’ conformations (**S1a** and **S1b**, together as **S1**) dominate over the ‘outside’ one (**S2**). Correspondingly, the PPi release from the inside to the outside is not energetically favored. Instead, the release is an activation process, and the rate-limiting step is the ‘jump’ transition from the inside to the outside. The mean first passage times (MFPTs) of the macrostate transitions were estimated ~ 0.2 μs with a standard error < 0.01 μs for **S1a** → **S1b**, and ~ 1.0 μs with a standard error < 0.1 μs for **S1b** → **S2** (the values vary slightly according to different numbers of microstates in the MSM construction, see Methods and **SI**), while the upper bounds of the MFPTs for **S1a** → **S1b** and **S1b** → **S2** were estimated at ~9 μs and ~ 20 μs, respectively (see Methods).

Next, for each macrostate, we analyzed the key residue interactions affecting the PPi release, by measuring the distance between each key residue and the PPi group. As shown in Fig 3C, three pairs of the distances, MgA-PPi, Asp812-MgB and Asp537-MgB, consistently increase from **S1a** to **S1b** and to **S2**, revealing the three-state character. In comparison, the distance Lys631-PPi increases and the corresponding association loosens only upon transition to **S2**, which demonstrate instead the two-state behavior. Interestingly, the distance Lys472-PPi gradually decreases so that the Lys472 association with PPi becomes comparatively stable in **S2**. In addition, the distance Arg627-PPi does not change obviously and the Arg627 association with PPi keeps moderate all along.

### The microsecond simulations suggest that the PPi release is not tightly coupled to the O-helix opening but is assisted by Lys472 swing

As noted above, the MSM is constructed based on extensive short MD simulations for nanoseconds each, which are individually too short to account for continuous slow motions. In order to examine better the slow motions in the PPi release, we conducted microsecond long MD simulations to the following systems: (i) The **unperturbed** PPi-bound product complex of T7 RNAP; (ii) A modified product complex with the stabilizing charges to PPi inside (Asp537, Asp812, and Lys631) turned off (**off-charge**); and (iii) Another modified product complex with PPi removed (**no PPi**). All simulations started from some **S1a** configurations. Later in the simulation, (i) was kept well in **S1a**, (ii) moved slightly toward **S1b** (see S1A and S1B Fig). Although no PPi release event was yet captured, we observed bending and some opening motions of the O-helix. Essentially, we also identified significant side chain swings of Lys472.

**Fig 4:**
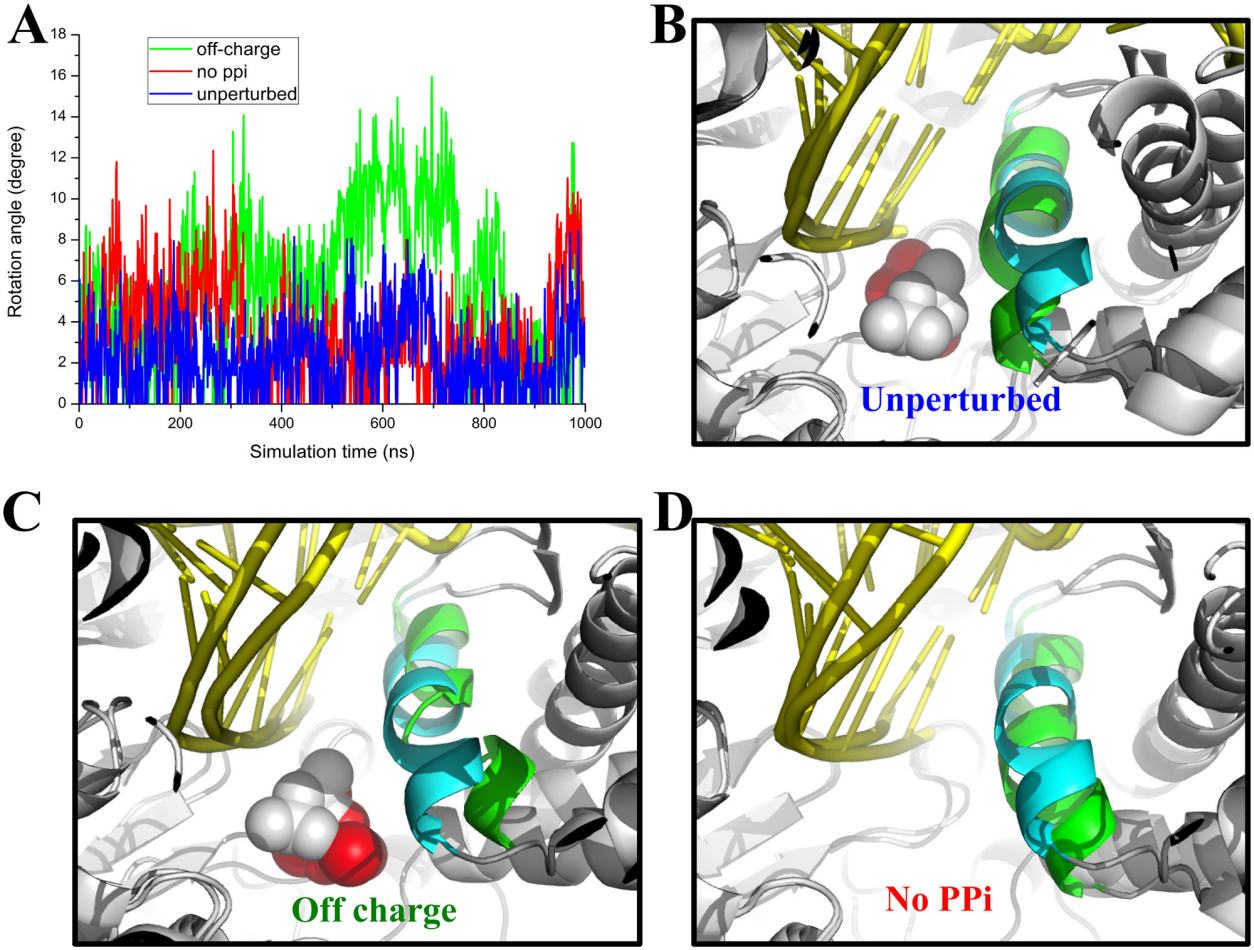
The O-helix rotation detected in microsecond MD simulations. (A) The rotation angles of the O-helix were measured from simulation (i), (ii) and (iii), and are shown in blue, green, and red, respectively. (B) The molecular snapshot view of the O-helix (green) taken from the unperturbed simulation (i). The initial closed form of the O-helix is also shown (cyan). (C and D) The molecular snapshots taken from the off-charge simulation (ii) and the no PPi simulation (iii), respectively, with the O-helix shown as in (B).

The O-helix is supposed to rotate to open (~ 22°) in the post-translocated configuration [50], while in the PPi-bound product complex and prior to the RNAP translocation, the O-helix is in a closed configuration [7]. The closed to open transition of the O-helix was suggested to couple the PPi release to the polymerase translocation in the PS model [7]. Accordingly, in our simulation, we wanted to know whether and how the O-helix switches from closed to open in response to the PPi release. To monitor opening motions of the O-helix, we recorded the overall rotation angles of the O-helix (see Fig 4), as well as the rotation angles measured from the N- and C-terminus of the O-helix (see S3 Fig), respectively. In general, we found that the overall rotation of the O-helix is smaller than that measured from the two terminus, which indicates occasional bending of the O-helix. Our analyses show that in the unperturbed simulation (i), the O-helix fluctuates near the closed configuration (< 10°, see Fig 4A and 4B). Occasionally, the N-term opens much more (up >15°, see S3B Fig) than the C-term, so that the helix bends significantly. The O-helix bending, however, imposed limited effects on the PPi group, which kept stable in the active site. Interestingly, in the off-charge simulation (ii), we noticed substantial openings of the O-helix (up > 15°, see Fig 4A and 4C), in particular, from the N-terminus (up > 20°, see S3C Fig). Nevertheless, PPi was still kept inside without significant movements, even though the PPi-Lys631 and PPi-Asp537/Asp812 distances largely increase (see S1D Fig). Hence, the PPi group does not seem to respond to the increased O-helix opening within our microsecond simulation. Moreover, we noticed that some non-charged local residues, including Gly538, Cys540, and Ser541, also stabilize PPi through forming hydrogen bonds or coordination with the MgB ion (see S4 Fig). These local interactions were present to stabilize PPi inside around the active site, in both the unperturbed simulation (i) and the off-charge simulation (ii). Finally, in the simulation (iii) with PPi removed, the O-helix gradually opened in the beginning of the simulation (up ~ 12°). The opening, however, was not sustained, as the rotation angle reduced (after ~ 300 ns) to that similar to the unperturbed simulation in the presence of PPi (see Fig 4A and 4D). Therefore, the O-helix could not open in response to the removal of PPi.

**Fig 5:**
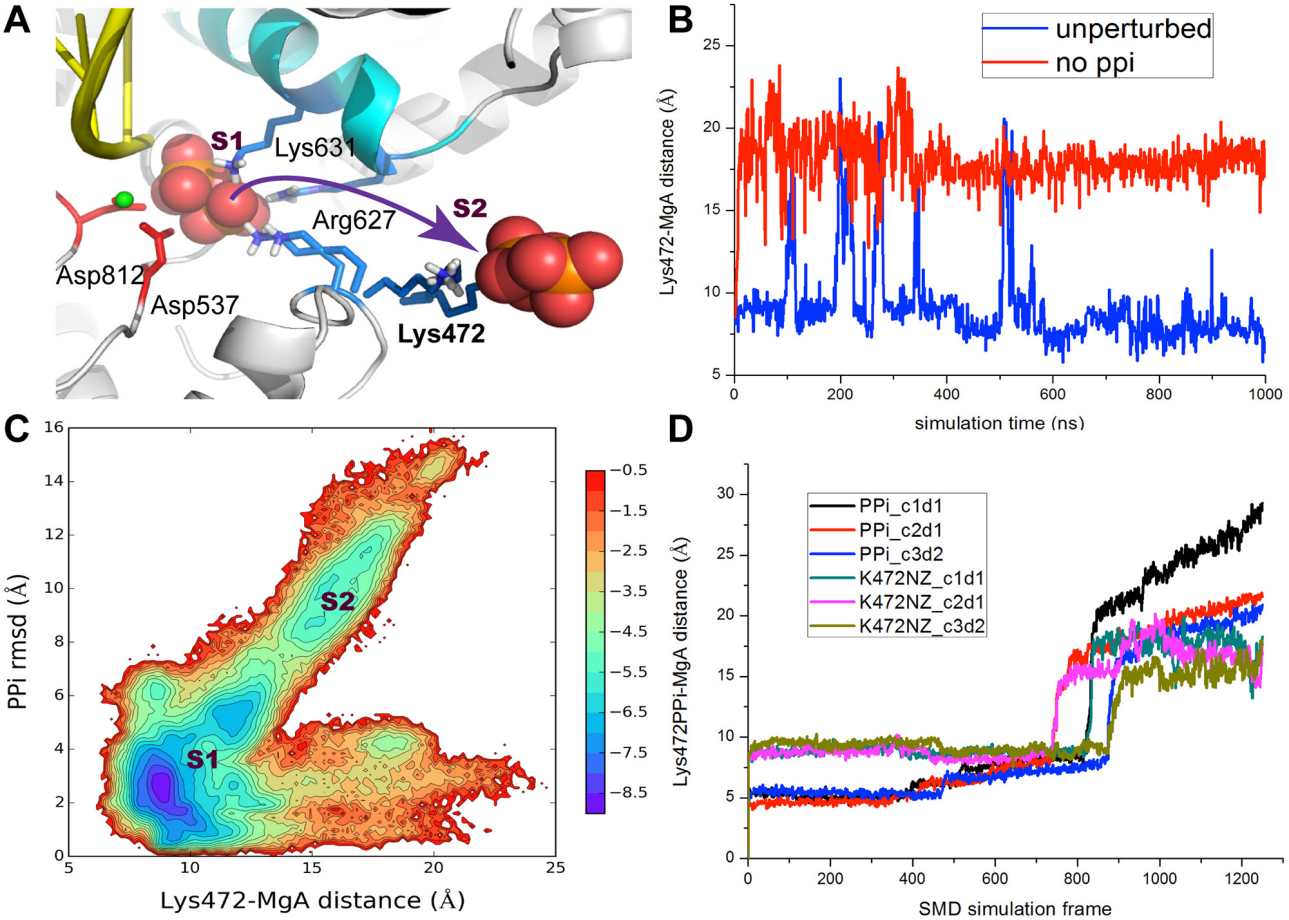
The side chain swing of Lys472 assists the PPi release. (A) The molecular view of Lys472 side chain that swings from pointing inside toward PPi in the active site (**S1**) to pointing outside to the solvent (**S2**). The two major configurations of the side chain were sampled from the long simulation (i and iii) and from the SMD simulations. (B) The Lys472-MgA distance measured from the long simulation (i) and (iii), with and without PPi, respectively. (C) The 2D density map (-ln P) generated from many short simulations used for the MSM construction. The map is depicted along the distance between Lys472 (NZ) and MgA and along the PPi RMSD. (D) The Lys472-MgA distance change along with the PPi-MgA distance change, obtained from three SMD simulations for nanoseconds, ran from different initial conditions/directions to pull PPi out.

On the other hand, we found dramatic swing motions of Lys472 that could substantially aid the PPi release. Lys472 is located on a flexible loop (residue 469 to 475) on the protein surface, which ensures that the Lys472 side chain can rotate freely. In Fig 5A, we show two major configurations of the Lys472 side chain, sampled from both the short SMD simulations and the microsecond long simulations (i and iii). In one configuration, the Lys472 side chain contacts on PPi and points inside toward the active site; in the other configuration, the side chain swings outward and points toward solvent. Indeed, in the first ~500 ns of the unperturbed long simulation (i), the Lys472 side chain kept close to PPi (in the active site) but occasionally, it swung away from PPi to point outward, at a frequency about once per 100 ns (see Fig 5B). In the latter part of the unperturbed simulation, the Lys472 side chain settled down and pointed only to the active site, grabbing PPi all along. In the off-charge simulation (ii), the Lys472 grabbed on PPi most of time, with only a couple of times, it swung away from PPi (not shown). In the absence of PPi in the simulation (iii), however, the Lys472 side chain swung away and relaxed to point outward, in response to the removal of PPi.

Notably, the two configurations of the Ly472 side chain correspond well to the inside and outside states **S1** (**S1a** and **S1b**) and **S2** from the MSM, respectively. As shown in Fig 5C, we projected all the PPi conformations from the short MD simulations onto two reaction coordinates: One is the PPi RMSD, and the other one is the distance between the Lys472-NZ atom and MgA. It is clearly seen that the swing motion of the Lys472 side chain correlates well with the PPi release. That is, the Lys472 points towards the active site in **S1** while it swings out to assist PPi to release toward **S2**. This high correlation was also observed in our SMD simulations. As shown in Fig 5D, in three PPi pulling events (with different initial conditions or pulling directions), we found that the Lys472 side chain swung along with PPi as PPi was pulled from the inside to the outside (see SMD S1 Movie in **SI**). Altogether with the above observations, we suggest that the side chain swing of Lys472 greatly promotes the PPi transition from **S1** to **S2**, or say, the PPi release.

## Discussion

The PPi release step is crucial to reveal the mechano-chemical coupling mechanism during the T7 RNAP transcription elongation. In this work we studied the process in structural dynamics detail by implementing atomistic MD simulations based on the high-resolution structure of T7 RNAP product complex. The conformational space of the PPi group along the releasing pathways was sampled through a large number of short (nanosecond) MD simulations as well as a small number of long (microsecond) MD simulations.

**Fig 6:**
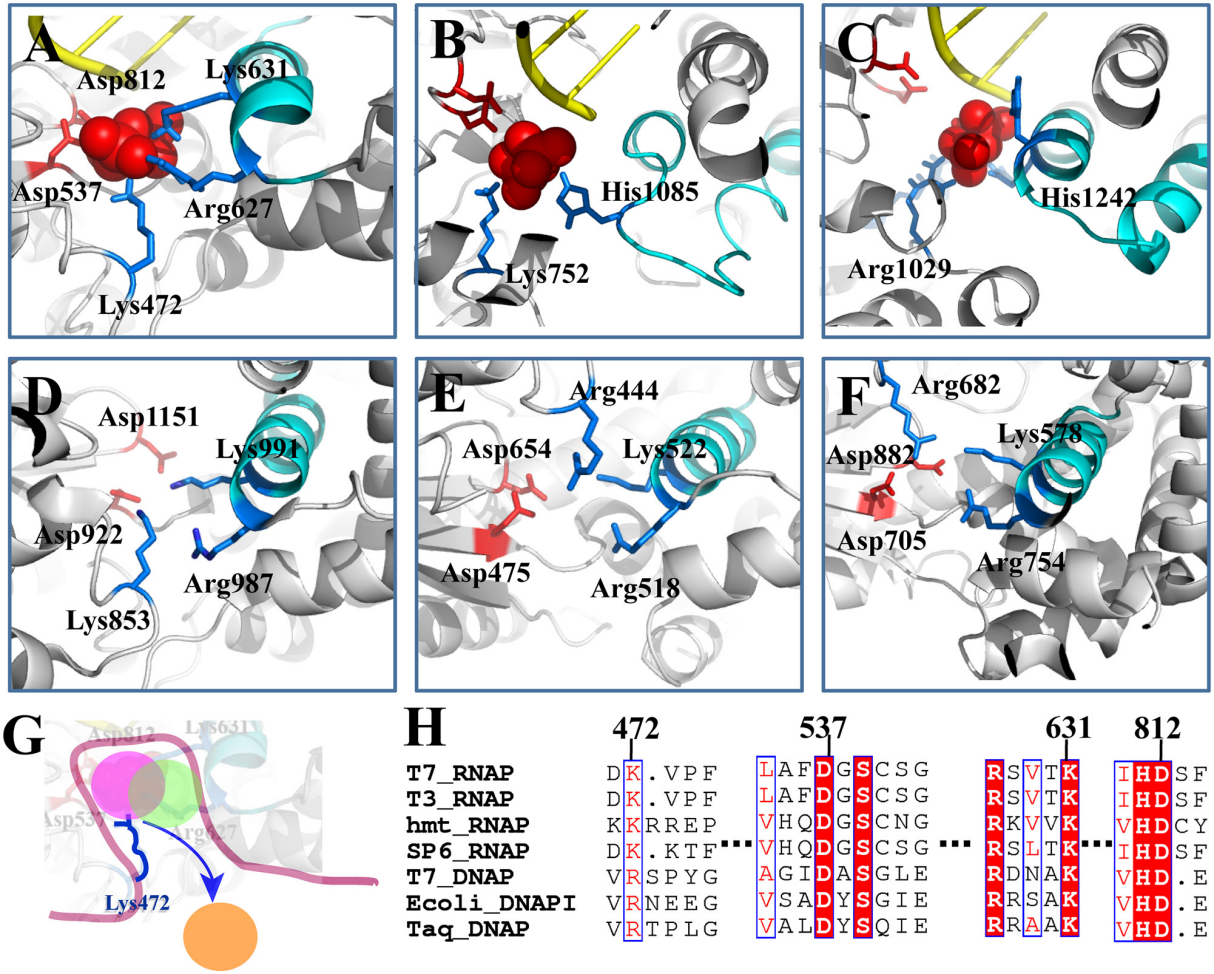
Comparing T7 RNAP with other RNAPs or DNAPs on the key PPi interactions around the active site. (A) T7 RNAP (PDB:1S77); (B) yeast Pol II [26]; (C) bacterial RNAP [25]; (D) human mitochondrial RNAP (PDB:3SPA); (E) T7 DNAP (PDB:1T7P); (F) E. coli DNAP I (PDB:1KLN). (G) A schematic of the jump from the cavity model, depicting the key lysine/arginine module that assists the PPi release from the active site (the colored disks represent the three meta-stable states as identified for T7 RNAP); (H) The sequence alignment of the single-subunit polymerases based on similarities of special sequence motifs and molecular structures. The essential charged residues for the PPi release are labeled. The conservations of corresponding residues in the sequence alignment are highlighted.

We constructed the MSM from those short MD simulations accumulated to about a couple of microseconds, which revealed a jump-from-cavity mechanism of the PPi release in T7 RNAP. Comparing the active site geometry of T7 RNAP with that of the multi-subunit RNAPs (see Figs 1 and 6), one can see a cavity-like structure in the former but a channel-alike one in the latter [25,26]. The structural differences lead to overall different PPi release behaviors in these two types of RNAPs. In the yeast RNA Pol II, it was indicated that the PPi release undergoes a four-state hopping mode whereby several positively charged residues in each hopping site facilitate the transfer of the PPi group between adjacent metastable states [26]. In the bacterial RNAP, a relatively shorter channel and an even faster dynamics of the PPi release comparing to that of Pol II was detected, with only two metastable states identified [25]. Notably, in the second metastable state (close to the outside), four positively charged residues are present closely to each other and greatly contribute to the stability of the PPi group. The transition from the inside to the outside, therefore, accompanies a slight free energy decrease due to the extra positive charges close to the outside, or say, it is electrostatically facilitated [25]. In contrast, in T7 RNAP, when PPi moves to the outside, only Lys472 and Arg627 marginally stabilize PPi. Indeed, the transition from the inside to the outside is hindered both sterically by local residues and electrostatically by those key charged residues, and thus requires a slight increase of the free energy. That says, the PPi release in T7 RNAP is a thermally activated process. Further dissociation of PPi from the protein surface site into the bulk water is not yet considered in current model. As mentioned early, the dissociation constant of PPi in T7 RNAP had been experimentally determined at ~ 1.2 mM [23]. A very low solution concentration of PPi, accordingly, would allow a close to irreversible transition of PPi into the bulk water.

When the PPi group (along with the MgB ion) initially locates inside around the active site, it forms close contact with the positively charged residue Lys631, as well as the two negatively charged residues Asp537 and Asp812 through MgB. These above contacts, however, disappear when the PPi-MgB group transfers to the **S2** site. In addition to those charged interactions, we also found several non-charged residues that can substantially stabilize the PPi-MgB group in the active site. In our controlled MD simulation with the charges of the above three residues (Lys631 and Asp537&812) turned off, the PPi dissociation from the active site still could not succeed within a microsecond. The close examination showed that several residues around the active site, including Gly538, Cys540, and Ser541, all contribute to the local PPi stabilization, by either forming hydrogen bonds with PPi or forming coordinated bond interactions with MgB (see S1 and S4 Figs). In brief, these non-charge local interactions contribute negatively to the activation process. The residues (from 538 to 541) are indeed conserved in the close species of viral single subunit RNAPs (see Fig 6H).

In the MSM construction, we estimated that the rate-limiting transition happens around a microsecond and up to tens of microseconds (see Methods and **Supporting Information**). Alternatively, using a trajectory mapping method for hierarchically clustering high dimensional time series [51], we identified equivalent slow modes of the release process and corresponding metastable states and time scales as that from the MSM (see Methods and **Supporting Information**). Previously, biochemical measurements on elongation kinetics of T7 RNAP showed that a slowest step (likely the finger domain or the O-helix closing) happens ~ 220 s^-1^ (~ 4 ms) prior to the catalysis, while the measurements indicated that the PPi release after the catalysis was much faster [23]. Hence, our model construction provides a reasonable estimation of the PPi release kinetic rate (micro to tens of microseconds) while elucidating the detailed structural mechanism.

Remarkably, we discovered that the side chain of Lys472 swings significantly to facilitate the jump-from-cavity transition of PPi. The tight coupling between the side chain swing and the PPi movement was directly captured and visualized in the SMD simulation (S1 Movie), in which PPi was pulled from the active site to bulk water within several nanoseconds. Additionally, samplings from a large number of unbiased MD simulations performed around the PPi pulling paths show that the Lys472-MgA distance is indeed a good reaction coordinate to describe the PPi release. Furthermore, in the microsecond simulations with and without PPi, the Lys472 side chain demonstrated clearly two-state behaviors: it pointed toward the active site and contacted PPi in one state, while it swung outside toward the bulk water in the other state, once PPi was removed. Hence, we suggest that Lys472 plays a significant role in the activation process of the PPi release. It can drag PPi out by the side chain swing, owning to the flexibility of the side chain and the loop the residue locates on. It is worth pointing out that the Lys472 side chain needs to settle down to tightly grab on PPi first to prepare for a successful swing to pull PPi out. The successful side chain swing of Lys472 appears to be a rare or rate-limiting event. It is thus expected that mutation of Lys472 to residues other than arginine would significantly slow down the PPi release process and possibly impact on the overall enzyme activities.

In this regard, we compared the T7 RNAP structure and protein sequence with that of other RNAPs and DNAPs (see Fig 6). According to the structural and sequence similarities [52,53], we find a conserved lysine residue from phage T3 and SP6 RNAPs, as well as from a human mitochondrial RNAP (Fig 6D and 6H), equivalent to Lys472 in T7 RNAP. Instead of lysine, however, an arginine residue in the corresponding position could be found in DNAPs (e.g. from phage T7 and bacteria species) that are structurally similar to T7 RNAP. Interestingly, the arginine side chain (e.g. Arg444 in T7 DNAP and Arg682 in *E*. *coli* DNAP I) approaches PPi from a ‘perpendicular’ direction relative to the Lys472 side chain positioning in T7 RNAP (see Fig 6E and 6F). On the other hand, the positively charged Lys631 and Arg627 located on the O-helix of T7 RNAP can always be found with their conserved counterparts in those single-subunit polymerases. Notably, the two catalytic aspartic acids (as Asp537 and 812 in T7 RNAP) are well conserved in all those polymerase species, in single or multi-subunit architecture [48,49]. Accordingly, we suggest that the PPi release mechanisms apply in all those structurally similar single-subunit RNAPs and DNAPs, i.e., start with the dissociation from the two catalytic aspartic acids, then proceed to a crucial jump-from-cavity activation and rate-limiting transition, which is assisted by a flexible lysine or arginine residue (see a schematic illustration in Fig 6G). We note that those non-charged local residues (538–541 in T7 RNAP) are conserved well within the viral RNAPs but not further for the rest of the single-subunit polymerases.

Surprisingly, one can also identify the lysine/arginine feature in similar locations around the active site in the multi-subunit RNAPs (see Fig 6B and 6C). In the yeast Pol II, there is a lysine residue Lys752 that can also grab on PPi to facilitate its hopping from the innermost state to the second metabstable state during the PPi release [26]. In the bacterial RNAP, one can also find an arginine residue Arg1029 playing a similar role in the PPi release [25]. Both the lysine and arginine residues locate opposite to the trigger loop, which unfolds/opens and folds/closes analogous to the O-helix in T7 RNAP. Hence, it seems that the lysine/arginine swing serves as a common module (see Fig 6G) to assist the PPi release in most of the polymerases, even though its role is shadowed by charge hopping characters in the multi-subunit RNAPs, due to their long release channel and abundent positive charges lying toward the exit of the channel to faciliate the PPi release.

Another important issue we examined is whether and how the PPi release couples with the O-helix opening in T7 RNAP. In our MD simulation of the unperturbed product complex over a microsecond, neither the PPi release nor the O-helix opening was yet detected. However, local or transient openings, in particular, the N-term bending of the O-helix, happened from time to time. Taken together, we see that neither the O-helix opening (in the off-charge simulation) induced the PPi release, nor the removal of the PPi (mimicking the full release) led to the O-helix opening in those microsecond simulations. Hence, the PPi release and the O-helix opening do not appear to be coupled tightly to each other. Considering that the translocation is accompanied by full opening of the O-helix, it is then unlikely that the PPi release could couple further to the translocation. Besides, the PPi release from the active site to the exit is found to be an activation process in T7 RNAP, without any free energy advantage. Therefore, the PPi release seems unable to energetically support the further O-helix opening and the translocation, as suggested by the power stroke mechanism. Note that there can still be free energy advantages after the PPi release from the protein surface, when the solution concentration of PPi is sufficiently low. In that case, PPi quickly diffuse into the bulk water once it dissociates from the protein, without being able to rebind to the RNAP.

### Conclusion

In this computational work, we examined structural dynamics of the PPi release process in the T7 RNAP transcription elongation at an atomistic resolution. We employed a large number of short MD simulations at nanoseconds to construct the MSM, combined with a small number of long simulations at microseconds to detect further slow essential motions. In contrast to the facilitated charge hopping mechanisms of the PPi release found in multi-subunit RNAPs, the PPi release in T7 RNAP is dominated by a jump-from-cavity activation process. Charge interactions from Lys631 on the O-helix and from two highly conserved aspartic acids Asp527 and 812 stabilize the PPi-MgB group inside around the active site to prevent the release. In addition, a group of non-charged residues interact locally with the PPi group to further hinder the activation process. Remarkably, a highly fluctuating lysine residue Lys472 grabs on PPi and pulls it out through significant side chain swings. The lysine and a similar arginine residue playing the same role can be found in other phage or mitochondria RNAPs, in structurally similar DNAPs, and even in the multi-subunit RNAPs. Hence, the flexible lysine/arginine side chain swing appears to be a common module to assist the PPi release, while the assisted jump-from-cavity mechanism of the PPi release may apply in general to a wide group of single subunit polymerases. Experimental mutations can be designed on those key residues to test their specific impacts. For example, turning off charge interactions individually from Lys631 and Lys472 is expected to accelerate and hinder the PPi release, respectively. Furthermore, the opening of the O-helix on the fingers domain does not appear to be tightly coupled to the PPi release according to our MD simulations, nor could the activated PPi release process energetically support the O-helix opening or further the translocation. Hence, current study favors the Brownian ratchet mechano-chemical coupling scenario over the power stroke one in the T7 RNAP elongation, consistent with single molecule studies revealing the Brownian ratchet nature of the RNAPs, no matter for single or multi-subunit species.

## Methods

### 1. MD simulation setup for the PPi-bound T7 RNAP complex

The crystal structure of the PPi-bound complex of T7 RNAP (PDB: 1S77) [7] was solvated with TIP3P water in a cubic box, and the minimum distance from the protein to the wall was set to 10Å. The RESP charges of the PPi group were calculated in AMBER package [54], after using the RED V software via HF/6-31G* method for the structure optimization [55]. The additional structural parameters were derived from the general Amber force field (GAFF) [56,57] (see SI S1 and S2 Tables). To neutralize the system and keep the ionic concentration of 0.1 M, 116 Na^+^ ions and 68 Cl^-^ ions were added. There were totally ~113,000 atoms in the final system. The MD simulations were performed using the GROMACS-4.6.5 software package [58]. The AMBER99sb force field with PARMBSC0 nucleic acid parameters was used to describe the model [59,60]. The cut off value for van der Waals (vdW) and short-range electrostatic interactions was set to 10 Å. Long-range electrostatic interactions were treated with the Particle-Mesh Ewald (PME) summation method [61]. The time-step was 2 fs and the neighbor list was updated every five steps. Firstly, the system was minimized using the steepest decent method for 50,000 steps, then 100 ps MD simulation under canonical ensemble was carried out. The temperature was set at 310 K using the velocity-rescaling thermostat [62]. After that, 100 ps MD simulation was run under NTP ensemble at 1 bar and 310 K, using the Parrinello-Rahman barostat [63,64] and the velocity rescaling thermostat, respectively. In the MD simulation, position restraints were imposed on the heavy atoms at the beginning of the simulation (for ~ 1 ns), and were then removed in the later equilibration. The above procedures were conducted to prepare equilibrated initial structures for the SMD simulations, to perform short MD simulations for the MSM construction, and to perform the microsecond long MD simulations.

### 2. Generating initial PPi release pathways using the Steered MD (SMD) simulations

We implemented the SMD simulations [65] to generate the initial PPi release pathways, as that were conducted previously [25,26]. To consider all possible PPi release pathways, the PPi-MgB group was pulled out of the active site along five directions (see **Supporting Information** and S5 Fig for details). The external force was applied to the center of mass of the PPi group with a force constant 10 kJ/mol/Å^2^ and the pulling rate was 0.01 Å/ps. During the SMD simulation, 100 kJ/mol/Å force restraints in the x, y, and z directions were imposed on protein Cα atoms and nucleic acid heavy atoms to maintain protein complex stability under the external force. For each pulling direction, three independent 3-ns SMD simulations were conducted (fifteen SMD simulations in total).

### 3. Seeding unbiased MD simulations to construct the Markov State Model (MSM)

The conformations obtained in the SMD simulations were grouped into 20 clusters using the K-center clustering algorithm [66]. In the clustering, the distance between a pair of conformations was set to the RMSD value of the PPi group (two phosphorus atoms and the bridged oxygen atom). The RMSD was computed by firstly aligning each conformation to one particular reference conformation where the PPi group locates in the active site right after the catalysis, according to the C_α_ atoms of the O-helix (627 to 640) on the finger domain. Then 3–5 conformations were randomly selected from each cluster (a total of 100 conformations) to conduct the following unbiased MD simulations. Each MD simulation was run for ~ 20 ns and the snapshots were saved every 2 ps. Altogether, an aggregation of ~2 μs simulations with 1000,000 conformations were obtained.

### 4. Construction the MSM for the PPi release

The Markov State Models (MSMs) describe molecular systems by partitioning the high-dimensional conformation space into a number of discrete metastable states [27–31], such that the conformational transitions within a certain metastable state are relatively fast comparing to the inter-state transitions. The disparate timescales allow constructing a markovian process where the probability of the transition from state *i* to state *j* (*T*_*ij*_) after a certain lag time *Δt* is only dependent on the current state *i* and not any former states. The *T*_*ij*_ for any two states *i* and *j* can be estimated from extensive short MD simulations with each state well equilibrated. Then by building a transition probability matrix ***T***, we can describe the stochastic transitions between different discrete states and propagate the markovian dynamics to a given long timescale:

$$P(n\Delta t)={\left[T(\Delta t)\right]}^{n}P(0)$$

where *P*(*nΔt*) is a vector of state populations at time *nΔt* and ***T*** is the transition probability matrix. Here we adopted a two-step procedure to construct the MSM for the PPi release: 1) Clustering the MD conformations into hundreds of microstates based on their geometric differences. 2) Lumping the microstates into several macrostates in order to visualize the key intermediate states during the PPi release [66]. The detailed procedures are described below.

1) Clustering the MD conformations into various numbers of microstates. Based on the geometric differences calculated from the RMSD of the PPi group (see the previous section), we clustered all the 1,000,000 MD conformations into 200, 300, 400 or 500 microstates using the K-center clustering algorithm implemented in the MSMBuilder software [67,68]. For each cluster size, we then constructed MSM and calculated the implied timescales at different lag times using:

$$\tau_{k}=-\frac{\tau }{\text{ln}\mu_{k}(\tau )}$$

where *μ*_*k*_ is the eigenvalue of the transition probability matrix ***T*** obtained in a certain lag time τ. Each implied time scale, representing an average transition time between two subsets of states, is one of the indicators to validate if the model is markovian [29]. For a sufficient long lag time τ, further increase of the lag time will not change the implied time scale when the model becomes markovian. As shown in S6A Fig, the implied timescales for different cluster sizes demonstrate similar kinetic behaviors for the slowest dynamics. Our result thus suggests that the MSM is robust to choices of different numbers of microstates, we therefore finally used one of the models, 200-state model, to further lump the microstates into macrostates and calculated the associated thermodynamic and kinetic properties. In the 200-state model, the average RMSD within each cluster was determined to be ~1.2 Å, indicating that the conformations within each cluster are geometrically similar to each other so that the kinetics within each cluster is relatively fast. The implied time scale curves level off after the lag time of 3 ns, indicating that the model becomes markovian. We therefore used the lag time of 3 ns as the markovian time to extract the thermodynamic and kinetic properties.

In order to further validate our MSM, we conducted the Chapman-Kolmogorov test [69]: we firstly selected the six most populated microstates, and for each state, we predicted its self-transition probability based on the transition probability matrix ***T*** generated at the markovian time of 3 ns. A good agreement is reached between the predicted values and the ones that are directly obtained from the MD simulations (see S6B Fig).

2) Visualizing key intermediates by lumping microstates into macrostates. We coarse-grained the microstates into a few macrostates in order to visualize the key intermediate states, which in turn, can help to elucidate specific mechanisms of the PPi release. Based on the 200-state model, we lumped the 200 microstates into three macrostates using the PCCA+ algorithm [70] implemented in the MSMBuilder. The equilibrium populations of the three macrostates and the MFPT of each macrostate-level transition were then calculated.

3) Calculating the Mean First Passage Time (MFPT). The MFPT for each pair of the states can be calculated using the following formula:

$$f_{ij}=\sum_{k}P_{ik}\times (t_{ik}+f_{kj})$$

Where *f*_ij_ is the MFPT for the transition from state *i* to state *j*, *P*_ik_ is the transition probability from state *i* to state *k* under a certain lag time *t*_ik_ (here *t*_ik_ is the markovian time and equals to 3 ns). Under the boundary condition of *f*_jj_ = 0, the *f*_ij_ can be determined by solving a set of linear equations. Based on the 200-state transition probability matrix constructed at the lag time of 3ns, we generated ten 10 ms long Monte Carlo (MC) trajectories. Then from each MC trajectory, we collected all the MFPT values for both the **S1a** → **S1b** and **S1b** → **S2** transitions and plotted the corresponding occurrence counts (see S7 Fig for the results of one of the MC trajectories). Next, we truncated those very small and very large MFPT values that correspond to the fastest and slowest dynamics, respectively. In such a way, we obtained a high-quality linear regression (see S7 Fig) according to the Poisson distribution. Upon that, we finally calculated the average of the MFPT for each macrostate transition. The average MFPTs were then obtained by averaging over the ten MC trajectories and the corresponding standard errors of the averages were calculated.

We also estimated the upper bound of the macrostate MFPT by obtaining the weighted sum of the microstate MFPTs without truncation. When the Markovian property is well maintained at the macrostate level, the estimation should provide a similar value as the average MFPT calculated above. However, in current case, as indicated above, the macrostate Markovian property is not well maintained without truncation (see S7A and S7B Fig). Consequently, the estimation here is dominated by the large microstate MFPTs, or say, by slow microstate transitions. In this way, the estimation gives an upper bound value of the MFPT for **S1a** → **S1b** at ~ 9 μs, and that for **S1b** → **S2** at ~ 20 μs.

Besides, partitioning the continuous phase space into a number of discrete states can result in losing the dynamic information within each discrete state. Therefore, using the Markov process to describe the stochastic motions of the biological system is an approximation to the continuous dynamics. We thus should take the errors caused by the discretization process into account when building the MSM. In order to evaluate the effects of the discretization errors on the MFPT, we calculated the MFPT values for other three models with different discretization resolutions (300, 400 and 500 microstates, respectively) by lumping the corresponding microstates into three macrostates. From S8 Fig, we can see that the MFPT values have very limited changes for different models, varying from 0.16 μs to 0.26 μs for the **S1a** → **S1b** transition and from 0.94 μs to 1.24 μs for **S1b** → **S2** transition, suggesting that the cluster size does not impact on the MFPT values significantly. It is also noted that although increasing number of microstates leads to smaller discretization errors, it also increases the statistic errors due to fewer transition counts between different states. We thus regard the 200-state model a reasonable one to build the MSM and extract the thermodynamic and kinetic properties, since the conformational space of each microstate is small enough to ensure a fast transition kinetics (the average RMSD comparing to the center conformation within each cluster was ~1.2Å).

### 5. Identifying slow modes and implied time scales using the trajectory mapping (TM) method

In order to confirm that the MSM construction above is robust using distance metrics other than the PPi RMSD, we utilized the trajectory mapping (TM) method [51] to find slow modes in the PPi release process. A total of 45 distances between oxygen or phosphorous atoms of PPi and Cα atoms of residues near the PPi release channel were used to identify configurations. After mapping the simulated trajectories, these distances are linearly combined to the principle components of the trajectory mapped vectors, which approximately correspond to slow variables in the PPi release process. As shown in S9 Fig, the first principle component (corresponding to the slowest variable) is closely correlated to the distance MgA-PPi (or Lys472-MgA), indicating the consistence between the MSM using the PPi RMSD and the TM analysis using the general distances. The implied time scale plots according to the first and first five principle components are also provided, showing similar results as that from the MSM.

## Supporting Information

S1 Text Descriptions on generating partial charges of PPi, calculating the O-helix rotation, and pulling PPi to generate five initial PPi release pathways. (PDF)

S1 Fig The sampling results from short and long simulations. (**A** & **B**) The 2-D population maps generated from the many short MD simulations, for the distance PPi-MgA (**A**) and D537-MgB (**B**) vs. the PPi RMSD, respectively. The configurations from the microsecond simulations to the unperturbed complex (black dots) and to the off-charge complex (gray dots) are shown on the map as well. (**C** & **D**) The distances from the charged key residues to PPi-MgB in both the unperturbed complex (**C**) and the off-charge complex (**D**).
(PDF)

S2 Fig Convergence tests on conformation samplings used for the MSM construction. Each population density profile was obtained as that in main Fig 2, projected along the PPi RMSD coordinate. The results were obtained from 100 trajectories, started from 5 to 20 ns (upper panel) and 10 to 20 ns (lower panel) for each individual trajectory. The convergence is improved from 10 to 20 ns around the **S1a**, **S1b**, and **S2** metastable states.
(PDF)

S3 Fig The O-helix rotation measured from both the N- and C-terminus. (**A**) The molecular view of the O-helix in different configurations are shown: An initial closed configuration (cyan), a random configuration picked from the unperturbed MD simulation (blue), and an open configuration (orange). The PPi-MgB group is shown in licorice. The rotation angles measured from the N-term (blue) and the C-term (red) were calculated from the long MD simulations, for the unperturbed product complex (**B**), the off-charge complex (**C**), and the product complex with PPi removed (**D**). The way calculating the O-helix rotation is illustrated in S1 text.
(PDF)

S4 Fig The local interactions that stabilize the (PP_i_-MgB)^2-^ group in the active site. (**A**) In addition to the residues Lys472, Arg627, Lys631, Asp537 and Asp812, several water molecules can enter into the active site and interact with the (PP_i_-MgB)^2-^ group. (**B**) Moreover, the backbone oxygen atom of residue Gly538 can coordinate with the MgB atom, and residues Cys540 and Ser541 can form hydrogen bonds with PPi through the N-H group of the amide bond. **(C & D)** The distances from the non-charged local residues Gly538, Cys540 and Ser541 to (PP_i_-MgB)^2-^, in both the unperturbed complex (**C**) and the off-charge complex (**D**).
(PDF)

S5 Fig Five pulling directions for generating the initial PPi release pathways using the SMD simulations. The pulling started from the initial position of PPi, and was made towards the COM of the Cα atoms of five different combinations of residues (see text), along *direction 1* (red arrow), *direction 2* (orange), *direction 3* (yellow), *direction 4* (green), and *direction 5* (blue). The polymerase is shown in surface representation and colored according to the charge of residues (blue: positive; red: negative; green, polar; white: nonpolar).
(PDF)

S6 Fig Validation of the MSM. (**A**) The implied timescales as the function of different lag time for the 200, 300, 400 and 500 microstate models, respectively. (**B**) For six most populated microstates, we predicted the self-transition probability for each state after a lag time of 3ns based on our 200-state MSM (blue line). The results were compared with the corresponding values counted directly from the MD simulations (red line).
(PDF)

S7 Fig Calculation of the MFPT from one of the ten MC long trajectories. Original MFPT counts (logarithm) in the MC trajectory for the **S1a** to **S1b** (**A**) and **S1b** to **S2** (**B**) transitions. Then, for each of the above two transitions, better linear regression was performed by truncating several MFPT values that correspond to either fastest or slowest dynamics (**C** and **D**, respectively).
(PDF)

S8 Fig The estimated MFPT for the S1a→S1b (A) and S1b→S2 (B) transitions under different number of microstates (200, 300, 400 and 500, respectively). For each model, the average and standard error of the MFPT were calculated by generating ten parallel 10 ms MC long trajectories that were built based on the corresponding transition probability matrix of MSM (see main Methods section for more details).
(PDF)

S9 Fig Using the trajectory mapping method to identify metastable states and implied time scales from the short simulation data. (**A**) The 2D population density maps (- lnP) along the slowest mode (B1) & the MgA-PPi distance (left), and along B1 & the Lys472-MgA distance (right). B1 appears to describe the PPi release equivalently well with the other two coordinates. Five metastable states reveal, with three of them within **S1a**, while the other two corresponding to **S1b** and **S2** respectively from the MSM (see S1A Fig). (**B**) The implied time scales calculated according to the slowest mode (B1; left) and according to the five slowest modes (B1 to 5; right).
(PDF)

S1 Table The calculated RESP charges for the PPi group. (XLSX)

S2 Table The structural parameters (for bond, angle, and dihedral) of the PPi group. * Pls refer to GROMACS (ref 58 in main) user manual.
(XLSX)

S1 Movie The movie was mode from one of the above SMD simulations (*direction 1* in S5 Fig), pulling PPi (in vdW spheres) from the inside to the outside in nanoseconds. In the movie, the nucleic acids are shown in yellow, the protein is shown in white, and the O-helix is highlighted in cyan. The three positively charged residue Lys631, Arg627, and Lys472 are shown in blue, the two negatively charged residue Asp537 and Asp812 are shown in red, and the two magnesium ions are in green. In particular, Lys472 is also highlighted in vdW spheres. The Lys472 side chain closely follows the PPi group, although the SMD force was only implemented on the PPi group but not on Lys472.
(MPG)

## References

[1] Buc H, Strick T, editors (2009) RNA polymerase as molecular motors. Cambridge, UK: The Royal Society of Chemistry.

[2] Borukhov S, Nudler E (2008) RNA polymerase: the vehicle of transcription. Trends in Microbiology 16: 126–134. 10.1016/j.tim.2007.12.006 18280161

[3] Bai L, Santangelo TJ, Wang MD (2006) SINGLE-MOLECULE ANALYSIS OF RNA POLYMERASE TRANSCRIPTION. Annual Review of Biophysics and Biomolecular Structure 35: 343–360. 16689640 10.1146/annurev.biophys.35.010406.150153

[4] Eid J, Fehr A, Gray J, Luong K, Lyle J, et al (2009) Real-Time DNA Sequencing from Single Polymerase Molecules. Science 323: 133–138. 10.1126/science.1162986 19023044

[5] Ronaghi M, Uhlén M, Nyrén P (1998) A Sequencing Method Based on Real-Time Pyrophosphate. Science 281: 363–365. 9705713 10.1126/science.281.5375.363

[6] Guo Q, Sousa R (2006) Translocation by T7 RNA Polymerase: A Sensitively Poised Brownian Ratchet. Journal of Molecular Biology 358: 241–254. 16516229 10.1016/j.jmb.2006.02.001

[7] Yin YW, Steitz TA (2004) The Structural Mechanism of Translocation and Helicase Activity in T7 RNA Polymerase. Cell 116: 393–404. 15016374 10.1016/s0092-8674(04)00120-5

[8] Wang H-Y, Elston T, Mogilner A, Oster G (1998) Force Generation in RNA Polymerase. Biophysical Journal 74: 1186–1202. 9512018 10.1016/S0006-3495(98)77834-8 PMC1299468

[9] Gelles J, Landick R (1998) RNA Polymerase as a Molecular Motor. Cell 93: 13–16. 9546386 10.1016/s0092-8674(00)81140-x

[10] Wang H, Oster G (2002) Ratchets, power strokes, and molecular motors. Applied Physics A 75: 315–323.

[11] Bustamante C, Keller D, Oster G (2001) The physics of molecular motors. Accounts of Chemical Research 34: 412–420. 11412078 10.1021/ar0001719

[12] Dangkulwanich M, Ishibashi T, Liu S, Kireeva ML, Lubkowska L, et al (2013) Complete dissection of transcription elongation reveals slow translocation of RNA polymerase II in a linear ratchet mechanism. eLIFE 2: e00971 10.7554/eLife.00971 24066225 PMC3778554

[13] Abbondanzieri EA, Greenleaf WJ, Shaevitz JW, Landick R, Block SM (2005) Direct observation of base-pair stepping by RNA polymerase. Nature 438: 460–465. 16284617 10.1038/nature04268 PMC1356566

[14] Thomen P, Lopez PJ, Heslot F (2005) Unravelling the Mechanism of RNA-Polymerase Forward Motion by Using Mechanical Force. Physical Review Letters 94: 128102 15903965 10.1103/PhysRevLett.94.128102

[15] Rong M, He B, McAllister WT, Durbin RK (1998) Promoter specificity determinants of T7 RNA polymerase. PNAS 95: 515–519. 9435223 10.1073/pnas.95.2.515 PMC18451

[16] Shis DL, Bennett MR (2014) Synthetic biology: the many facets of T7 RNA polymerase. Molecular Systems Biology 10: n/a–n/a. 10.15252/msb.20145492 PMC4299499 25080495

[17] Citorik RJ, Mimee M, Lu TK (2014) Bacteriophage-based synthetic biology for the study of infectious diseases. Current Opinion in Microbiology 19: 59–69. 10.1016/j.mib.2014.05.022 24997401 PMC4125527

[18] Steitz TA (2009) The structural changes of T7 RNA polymerase from transcription initiation to elongation. Current Opinion in Structural Biology 19: 683–690. 10.1016/j.sbi.2009.09.001 19811903 PMC2818687

[19] Temiakov D, Patlan V, Anikin M, McAllister WT, Yokoyama S, et al (2004) Structural Basis for Substrate Selection by T7 RNA Polymerase. Cell 116: 381–391. 15016373 10.1016/s0092-8674(04)00059-5

[20] Thomen P, Lopez PJ, Bockelmann U, Guillerez J, Dreyfus M, et al (2008) T7 RNA Polymerase Studied by Force Measurements Varying Cofactor Concentration. Biophysical Journal 95: 2423–2433. 10.1529/biophysj.107.125096 18708471 PMC2517023

[21] Kim JH, Larson RG (2007) Single-molecule analysis of 1D diffusion and transcription elongation of T7 RNA polymerase along individual stretched DNA molecules. Nucleic Acids Research: gkm332. 10.1093/nar/gkm332 PMC1920259 17526520

[22] Tang G-Q, Roy R, Bandwar RP, Ha T, Patel SS (2009) Real-time observation of the transition from transcription initiation to elongation of the RNA polymerase. Proceedings of the National Academy of Sciences 106: 22175–22180. 10.1073/pnas.0906979106 PMC2799739 20018723

[23] Anand VS, Patel SS (2006) Transient State Kinetics of Transcription Elongation by T7 RNA Polymerase. The jouornal of biological chemistry 281: 35677–35685. 10.1074/jbc.M608180200 17005565

[24] Sousa R, Mukherjee S, Kivie M (2003) T7 RNA Polymerase Progress in Nucleic Acid Research and Molecular Biology: Academic Press pp. 1–41. 10.1016/s0079-6603(03)01001-8 12882513

[25] Da L-T, Pardo A, F., Wang D, Huang X (2013) A Two-State Model for the Dynamics of the Pyrophosphate Ion Release in Bacterial RNA Polymerase. PloS Computational Biology 9: e1003020 10.1371/journal.pcbi.1003020 23592966 PMC3617016

[26] Da L, Wang D, Huang X (2011) Dynamics of Pyrophosphate Ion Release and Its Coupled Trigger Loop Motion from Closed to Open State in RNA Polymerase II. Journal of the American Chemical Society 134: 2399–2406. 10.1021/ja210656k PMC3273452 22206270

[27] Da L-T, Sheong F, Silva D-A, Huang X (2014) Application of Markov State Models to Simulate Long Timescale Dynamics of Biological Macromolecules. In: Han K-l, Zhang X, Yang M-j, editors. Protein Conformational Dynamics: Springer International Publishing. pp. 29–66. 10.1007/978-3-319-02970-2_2 24446356

[28] Chodera JD, Noé F (2014) Markov state models of biomolecular conformational dynamics. Current Opinion in Structural Biology 25: 135–144. 10.1016/j.sbi.2014.04.002 24836551 PMC4124001

[29] Pande VS, Beauchamp K, Bowman GR (2010) Everything you wanted to know about Markov State Models but were afraid to ask. Methods (San Diego, Calif) 52: 99–105. 10.1016/j.ymeth.2010.06.002 PMC2933958 20570730

[30] Noe F, Fischer S (2008) Transition networks for modeling the kinetics of conformational change in macromolecules. Curr Opin Struct Biol 18: 154–162. 10.1016/j.sbi.2008.01.008 18378442

[31] Chodera JD, Singhal N, Pande VS, Dill KA, Swope WC (2007) Automatic discovery of metastable states for the construction of Markov models of macromolecular conformational dynamics. J Chem Phys 126: 155101 17461665 10.1063/1.2714538

[32] Zhuang W, Cui RZ, Silva DA, Huang X (2011) Simulating the T-jump-triggered unfolding dynamics of trpzip2 peptide and its time-resolved IR and two-dimensional IR signals using the Markov state model approach. J Phys Chem B 115: 5415–5424. 10.1021/jp109592b 21388153

[33] Lane T, Bowman G, Beauchamp K, Voelz V, Pande V (2011) Markov State Model Reveals Folding and Functional Dynamics in Ultra-Long MD Trajectories. Journal of the American Chemical Society 133: 18413–18419. 10.1021/ja207470h 21988563 PMC3227799

[34] Huang X, Bowman GR, Bacallado S, Pande VS (2009) Rapid equilibrium sampling initiated from nonequilibrium data. PNAS 106: 19765–19769. 10.1073/pnas.0909088106 19805023 PMC2785240

[35] Kohlhoff KJ, Shukla D, Lawrenz M, Bowman GR, Konerding DE, et al (2014) Cloud-based simulations on Google Exacycle reveal ligand modulation of GPCR activation pathways. Nat Chem 6: 15–21. 10.1038/nchem.1821 24345941 PMC3923464

[36] Silva DA, Bowman GR, Sosa-Peinado A, Huang X (2011) A Role for Both Conformational Selection and Induced Fit in Ligand Binding by the LAO Protein. PLos Computational Biology 7: e1002054 10.1371/journal.pcbi.1002054 21637799 PMC3102756

[37] Choudhary OP, Paz A, Adelman JL, Colletier JP, Abramson J, et al (2014) Structure-guided simulations illuminate the mechanism of ATP transport through VDAC1. Nat Struct Mol Biol 21: 626–632. 10.1038/nsmb.2841 24908397 PMC4157756

[38] Silva DA, Weiss DR, Pardo Avila F, Da LT, Levitt M, et al (2014) Millisecond dynamics of RNA polymerase II translocation at atomic resolution. Proc Natl Acad Sci U S A 111: 7665–7670. 10.1073/pnas.1315751111 24753580 PMC4040580

[39] Fouqueau T, Zeller ME, Cheung AC, Cramer P, Thomm M (2013) The RNA polymerase trigger loop functions in all three phases of the transcription cycle. Nucleic Acids Research 41: 7048–7059. 10.1093/nar/gkt433 23737452 PMC3737540

[40] Toulokhonov I, Zhang J, Palangat M, Landick R (2007) A Central Role of the RNA Polymerase Trigger Loop in Active-Site Rearrangement during Transcriptional Pausing. Molecular Cell 27: 406–419. 17679091 10.1016/j.molcel.2007.06.008

[41] Miller BR III, Parish CA, Wu EY (2014) Molecular Dynamics Study of the Opening Mechanism for DNA Polymerase I. PLos Computational Biology 10: e1003961 10.1371/journal.pcbi.1003961 25474643 PMC4256020

[42] Duan B, Wu S, Da L-T, Yu J (2014) A Critical Residue Selectively Recruits Nucleotides for T7 RNA Polymerase Transcription Fidelity Control. Biophysical Journal 107: 2130–2140. 10.1016/j.bpj.2014.09.038 25418098 PMC4223216

[43] Yu J, Oster G (2012) A Small Post-translocation Energy Bias Aids Nucleotide Selection in T7 RNA Polymerase Transcription. Biophysical Journal 102: 532–541. 10.1016/j.bpj.2011.12.028 22325276 PMC3274829

[44] Golosov A, Warren J, Beese L, Karplus M (2010) The Mechanism of the Translocation Step in DNA Replication by DNA Polymerase I: A Computer Simulation Analysis. Structure 18: 83–93. 10.1016/j.str.2009.10.014 20152155 PMC3325112

[45] Woo H-J, Liu Y, Sousa R (2008) Molecular dynamics studies of the energetics of translocation in model T7 RNA polymerase elongation complexes. Proteins: Structure, Function, and Bioinformatics 73: 1021–1036. 10.1002/prot.22134 PMC4447105 18536012

[46] Florián J, Goodman MF, Warshel A (2005) Computer simulations of protein functions: Searching for the molecular origin of the replication fidelity of DNA polymerases. Proceedings of the National Academy of Sciences of the United States of America 102: 6819–6824. 15863620 10.1073/pnas.0408173102 PMC1100748

[47] Schlick T, Arora K, Beard WA, Wilson SH (2012) Perspective: pre-chemistry conformational changes in DNA polymerase mechanisms. Theoretcial Chemistry Accounts 131: 1287. 10.1007/s00214-012-1287-7 PMC3583561 23459563

[48] Steitz TA (1999) DNA Polymerases: Structural Diversity and Common Mechanisms. Journal of Biological Chemistry 274: 17395–17398. 10364165 10.1074/jbc.274.25.17395

[49] Sosunov V, Zorov S, Sosunova E, Nikolaev A, Zakeyeva I, et al (2005) The involvement of the aspartate triad of the active center in all catalytic activities of multisubunit RNA polymerase. Nucleic Acids Research 33: 4202–4211. 16049026 10.1093/nar/gki688 PMC1180743

[50] Yin YW, Steitz TA (2002) Structural Basis for the Transition from Initiation to Elongation Transcription in T7 RNA Polymerase. Science 298: 1387–1395. 12242451 10.1126/science.1077464

[51] Gong L, Zhou X, Ouyang Z (2015) Systematically Constructing Kinetic Transition Network in Polypeptide from Top to Down: Trajectory Mapping. PLos One 10: e0125932 10.1371/journal.pone.0125932 25962177 PMC4427365

[52] McWilliam H, Li W, Uludag M, Squizzato S, Park YM, et al (2013) Analysis Tool Web Services from the EMBL-EBI. Nucleic acids research 41: W597–600. 10.1093/nar/gkt376 23671338 PMC3692137

[53] Eargle J, Wright D, Luthey-Schulten Z (2006) Multiple Alignment of protein structures and sequences for VMD. Bioinformatics 22: 504–506. 16339280 10.1093/bioinformatics/bti825

[54] Salomon-Ferrer R, Case DA, Walker RC (2012) An overview of the Amber biomolecular simulation package. Wiley Interdisciplinary Reviews: Computational Molecular Science 3: 198–210.

[55] Dupradeau FY, Pigache A, Zaffran T, Savineau C, Lelong R, et al (2010) The R.E.D. tools: advances in RESP and ESP charge derivation and force field library building. Phys Chem Chem Phys 12: 7821–7839. 10.1039/c0cp00111b 20574571 PMC2918240

[56] Wang J, Wang W, Kollman PA, Case DA (2006) Automatic atom type and bond type perception in molecular mechanical calculations. Journal of Molecular Graphics and Modelling 25: 247260. 10.1016/j.jmgm.2005.12.005 16458552

[57] Wang J, Wolf RM, Caldwell JW, Kollman PA, Case DA (2004) Development and testing of a general AMBER force field. Journal of Computational Chemistry 25: 1157–1174. 15116359 10.1002/jcc.20035

[58] Hess B, Kutzner C, van der Spoel D, Lindahl E (2008) GROMACS 4: Algorithms for Highly Efficient, Load-Balanced, and Scalable Molecular Simulation. Journal of Chemical Theory and Computation 4: 435–447. 26620784 10.1021/ct700301q

[59] Hornak V, Abel R, Okur A, Strockbine B, Roitberg A, et al (2006) Comparison of multiple Amber force fields and development of improved protein backbone parameters. Proteins: Structure, Function, and Bioinformatics 65: 712–725. 10.1002/prot.21123 PMC4805110 16981200

[60] Joung IS, Cheatham TE (2009) Molecular Dynamics Simulations of the Dynamic and Energetic Properties of Alkali and Halide Ions Using Water-Model-Specific Ion Parameters. The Journal of Physical Chemistry B 113: 13279–13290. 10.1021/jp902584c 19757835 PMC2755304

[61] Essmann U, Perera L, Berkowitz ML, Darden T, Lee H, et al (1995) A smooth particle mesh Ewald method. The Journal of Chemical Physics 103: 8577–8593.

[62] Bussi G, Donadio D, Parrinello M (2007) Canonical sampling through velocity rescaling. The Journal of Chemical Physics 126: -. 10.1063/1.2408420 17212484

[63] Parrinello M, Rahman A (1981) Polymorphic transitions in single crystals: A new molecular dynamics method. Journal of Applied Physics 52: 7182–7190.

[64] Nosé S, Klein ML (1983) Constant pressure molecular dynamics for molecular systems. Molecular Physics 50: 1055–1076.

[65] Isralewitz B, Gao M, Schulten K (2001) Steered molecular dynamics and mechanical functions of proteins. Current Opinion in Structural Biology 11: 224–230. 11297932 10.1016/s0959-440x(00)00194-9

[66] Bowman GR, Huang X, Pande VS (2009) Using generalized ensemble simulations and Markov state models to identify conformational states. Methods 49: 197–201. 10.1016/j.ymeth.2009.04.013 19410002 PMC2753735

[67] Beauchamp KA, Bowman GR, Lane TJ, Maibaum L, Haque IS, et al (2011) MSMBuilder2: Modeling Conformational Dynamics at the Picosecond to Millisecond Scale. Journal of chemical theory and computation 7: 3412–3419. 22125474 10.1021/ct200463m PMC3224091

[68] Bowman G (2014) A Tutorial on Building Markov State Models with MSMBuilder and Coarse-Graining Them with BACE In: Livesay DR, editor. Protein Dynamics: Humana Press pp. 141–158. 10.1007/978-1-62703-658-0_8 24061920

[69] Noé F, Schütte C, Vanden-Eijnden E, Reich L, Weikl TR (2009) Constructing the equilibrium ensemble of folding pathways from short off-equilibrium simulations. Proceedings of the National Academy of Sciences 106: 19011–19016. 10.1073/pnas.0905466106 PMC2772816 19887634

[70] Deuflhard P, Weber M (2005) Robust Perron cluster analysis in conformation dynamics. Linear Algebra Appl 398: 161–184.

