# Supplementary material for: A Jump-from-Cavity Pyrophosphate Ion Release Assisted by a Key Lysine Residue in T7 RNA Polymerase Transcription Elongation
Source: PLoS Comput Biol. 2015 Nov 24;11(11):e1004624. doi: 10.1371/journal.pcbi.1004624 (PMC4658072; doi:10.1371/journal.pcbi.1004624)
Supplement: S1 Text — (PDF) [file pcbi.1004624.s001.pdf]

Supporting Information Text

Generating partial charges of PPi

We used R.E.D. V software with HF/6-31G* method (ref 55 in main **Methods**) to optimize the PPi structure and then used RESP program implemented in AMBER package to obtain the partial charges. Other structural parameters, including the bond, angle, and dihedral, were derived from the general AMBER force field (GAFF). The charge and structural parameters of PPi are provided below in **S1 Table** and **S2 Table**. Note that the QM optimization method and the basis sets we chose are the same as that used in the previous study e.g., from Meagher et al, J. Comp. Chem. 2003.

In our current study, we did not treat the $(PPi\text{-}MgB)^{2-}$ as one group for the structural optimization as that was conducted previously (Da et al JACS 2011, ref 26 in main), since the MgB ion is located at a position deviated from PPi in the T7 RNAP crystal structure (PDB: 1S77). As we optimized the PPi structure separately from MgB and obtained partial charges (in **S1 Table**), MgB is simply assigned with a charge of +2.0 in our case. To test how the PPi-MgB dynamics possibly varies under alternative parameter sets such as that used in Da's 2011 work (i.e., optimizing structure of PPi and MgB together), we had performed a test MD simulation adopting those parameters (Da et al JACS, 2011). Our results show that the interactions between the PPi-MgB group and its surrounding residues were less stable in the initial stage of the test MD simulation compared to our original simulations, which is due to the lower partial charge of the MgB ion (+1.4) implemented in the test run. Nevertheless, when we extended the test MD simulation to ~200 ns, we found that the PPi association with most of nearby residues were similar to that in our original unperturbed MD simulation, and PPi also kept stable within a same coordinate subspace as that in the original simulation, e.g., according to the PPi RMSD and PPi-MgA distance. Therefore, the most essential dynamics of PPi appear to be preserved upon slight variations of the PPi-MgB parameters.

Calculating the O-helix rotation

To measure the O-helix (residue from 627 to 640) rotation relative to the initial closed product configuration, the MD simulation structural snapshots were aligned to the product crystal structure (PDB: 1S77) according to the palm domain. The orientation of the O-helix is determined through a vector $\boldsymbol{v}$ linking the center of the mass (COM) of the N-term (627 to 633) with that of the C-term (634 to 640). A reference plane was defined by the O-helix orientation in the closed state $\boldsymbol{v}^{\mathbf{c}}$ and that in the post-translocation (PDB 1MSW) open state $\boldsymbol{v}^{\mathbf{o}}$, as being perpendicular to the normal vector $\boldsymbol{v}^{\mathbf{c}} \times \boldsymbol{v}^{\mathbf{o}}$. The orientation vector $\boldsymbol{v}$ is accordingly projected into the reference plane as $\boldsymbol{v}^{\perp}$, and the O-helix rotation is measured as the angle between $\boldsymbol{v}^{\mathbf{c}}$ and $\boldsymbol{v}^{\perp}$. When the O-helix rotation was measured from the N/C-terminal side, the orientation vector $\boldsymbol{v}$ was then determined by linking the COM of the N/C-term with that of the O-helix overall, followed by similar procedures above.

Pulling PPi to generate five initial PPi release pathways

In order to obtain the initial PPi release pathway, we implemented SMD simulations to pull the PPi-MgB group out of the active site. The pulling was performed along five directions determined by the COMs of five combinations of residue groups. Based on visualizing the charge distributions on the protein, residue 482-486, 503-507, 619-623 and 661-665 were selected as group I, II, III and IV, respectively. Then the five pulling directions were conducted towards the COM of the Cα atoms of group I and III (*direction 1*), then toward that of group I and IV (*direction 2*), of group II and IV (*direction 3*), of group II and III (*direction 4*), and that of four groups together (*direction 5*). See **S5 Fig** for a molecular view of the five pulling directions.
