# Supplementary figures and images for: A Jump-from-Cavity Pyrophosphate Ion Release Assisted by a Key Lysine Residue in T7 RNA Polymerase Transcription Elongation
Source: PLoS Comput Biol. 2015 Nov 24;11(11):e1004624. doi: 10.1371/journal.pcbi.1004624 (PMC4658072; doi:10.1371/journal.pcbi.1004624)

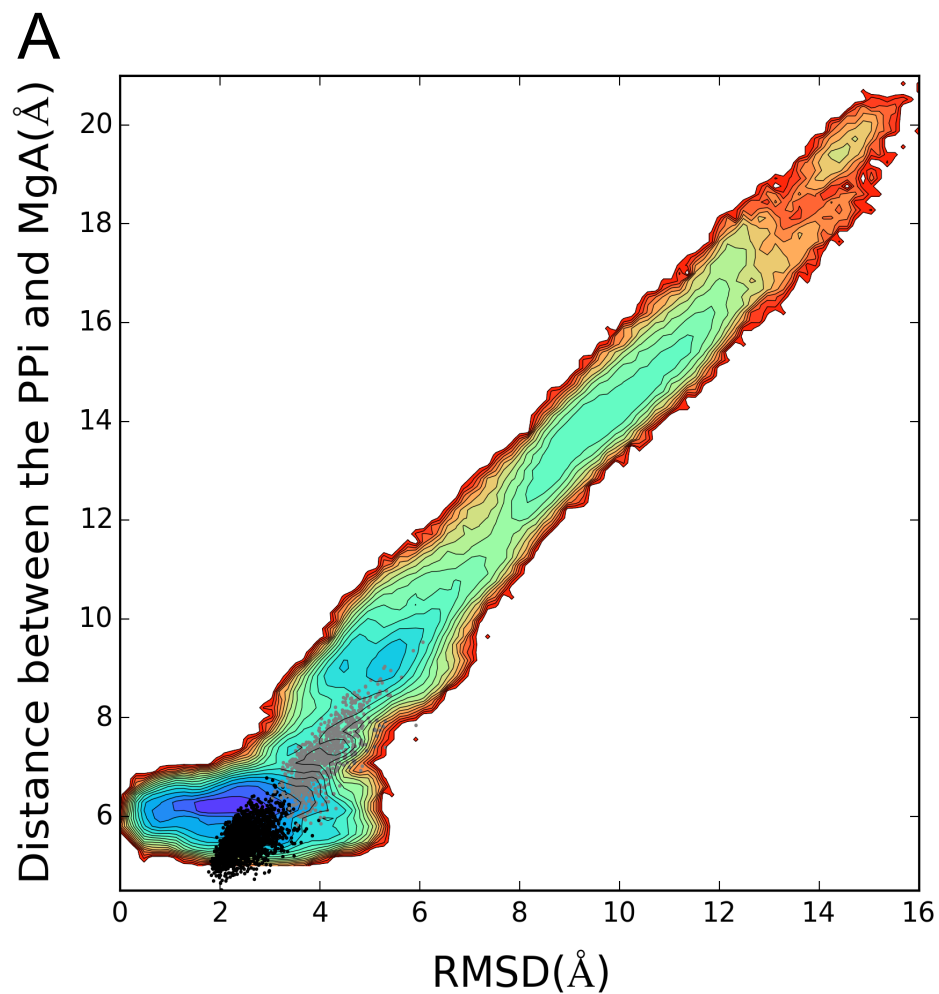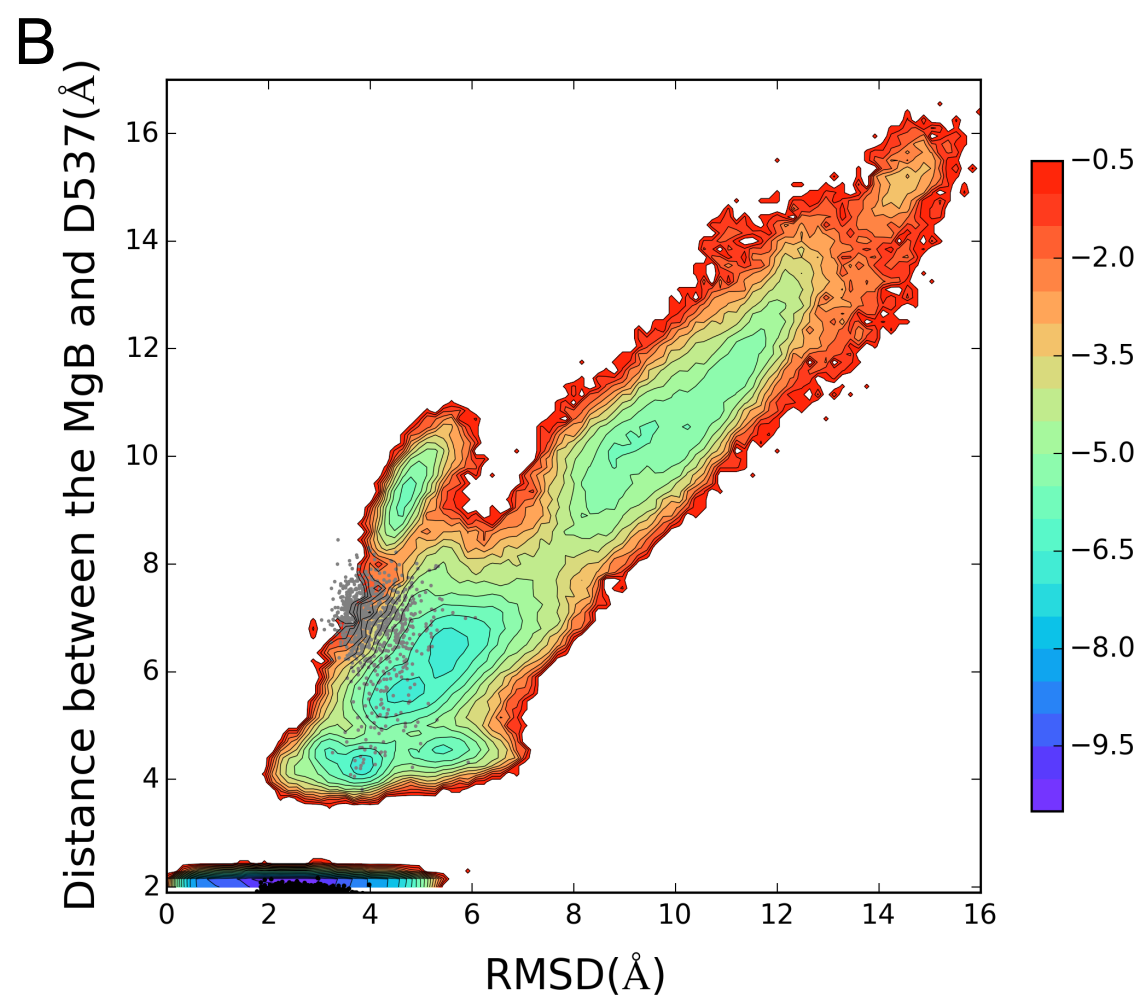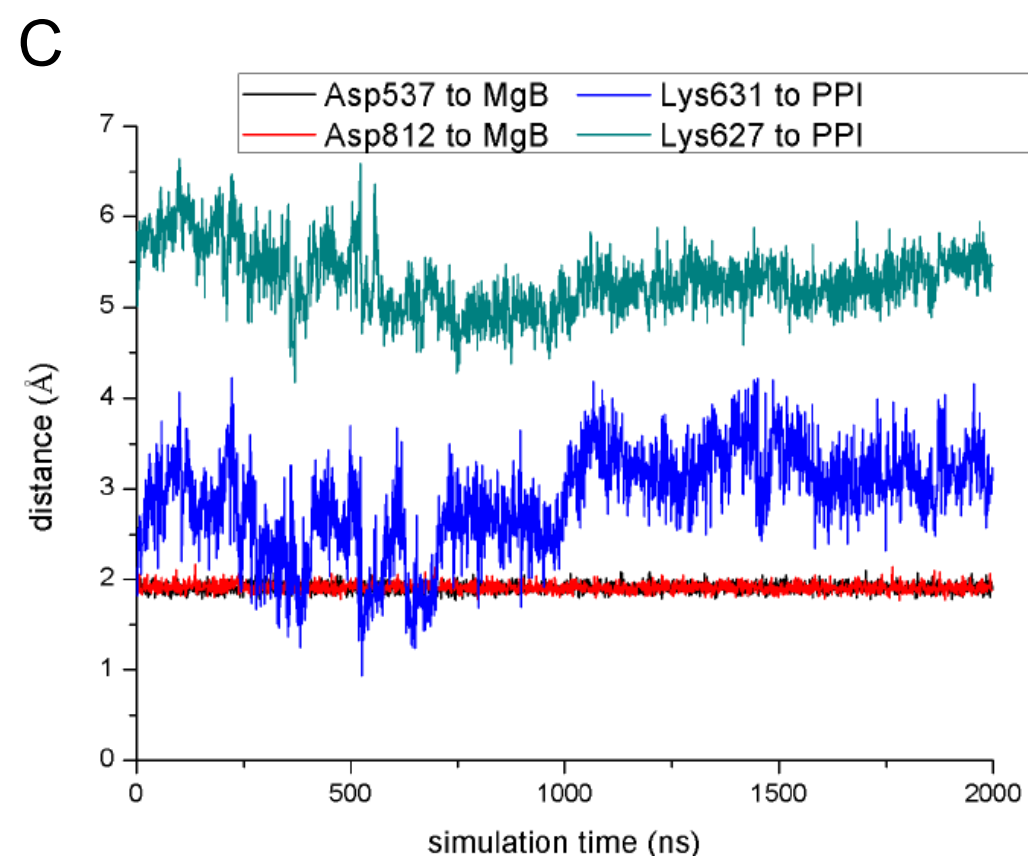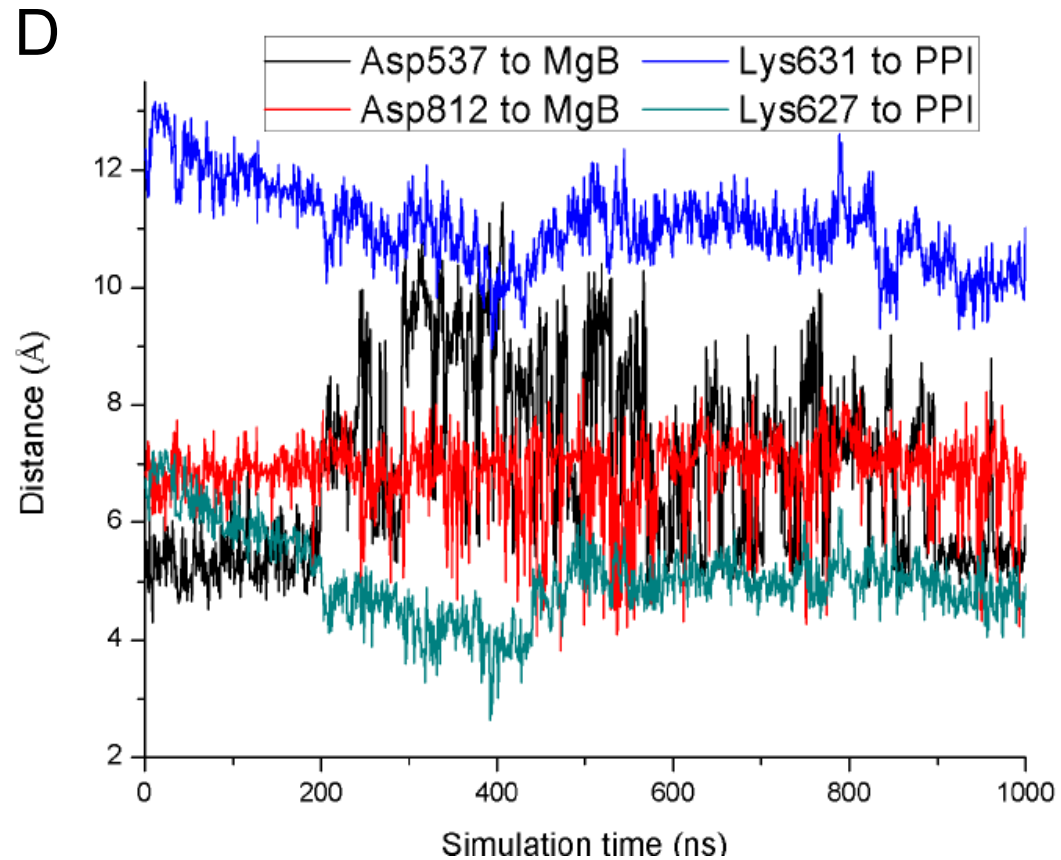

Supplement: S1 Fig — (A & B) The 2-D population maps generated from the many short MD simulations, for the distance PPi-MgA (A) and D537-MgB (B) vs. the PPi RMSD, respectively. The configurations from the microsecond simulations to the unperturbed complex (black dots) and to the off-charge complex (gray dots) are shown on the map as well. (C & D) The distances from the charged key residues to PPi-MgB in both the unperturbed complex (C) and the off-charge complex (D). (PDF) [file pcbi.1004624.s002.pdf]

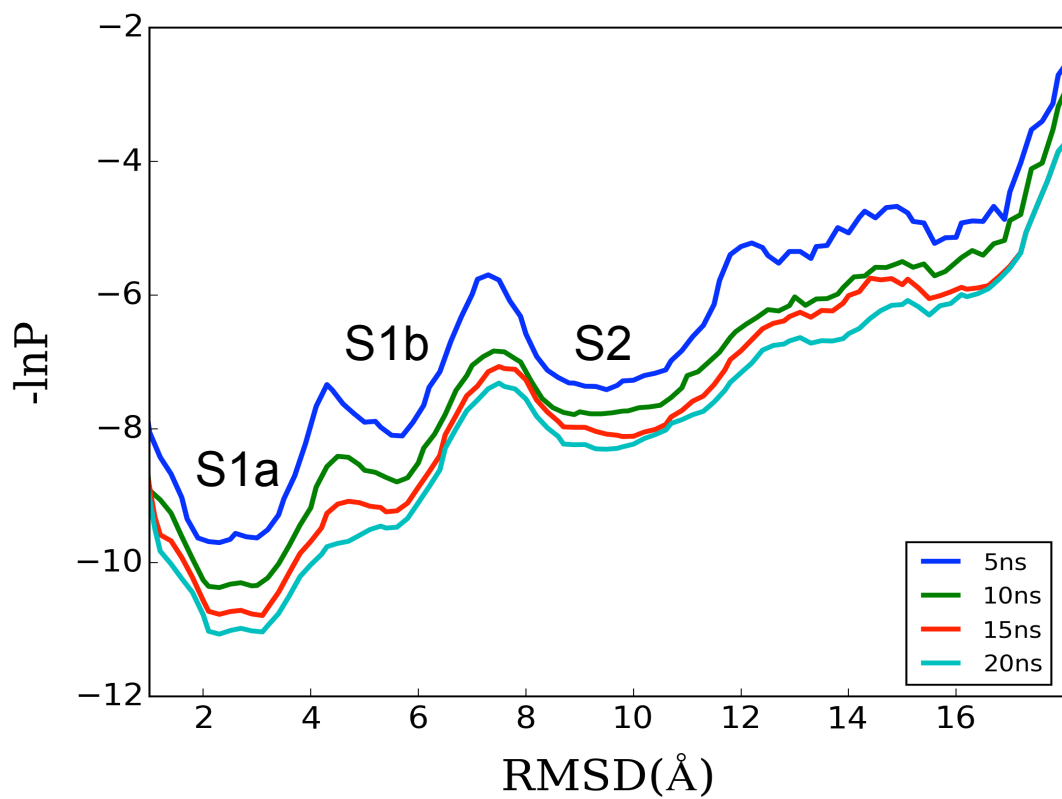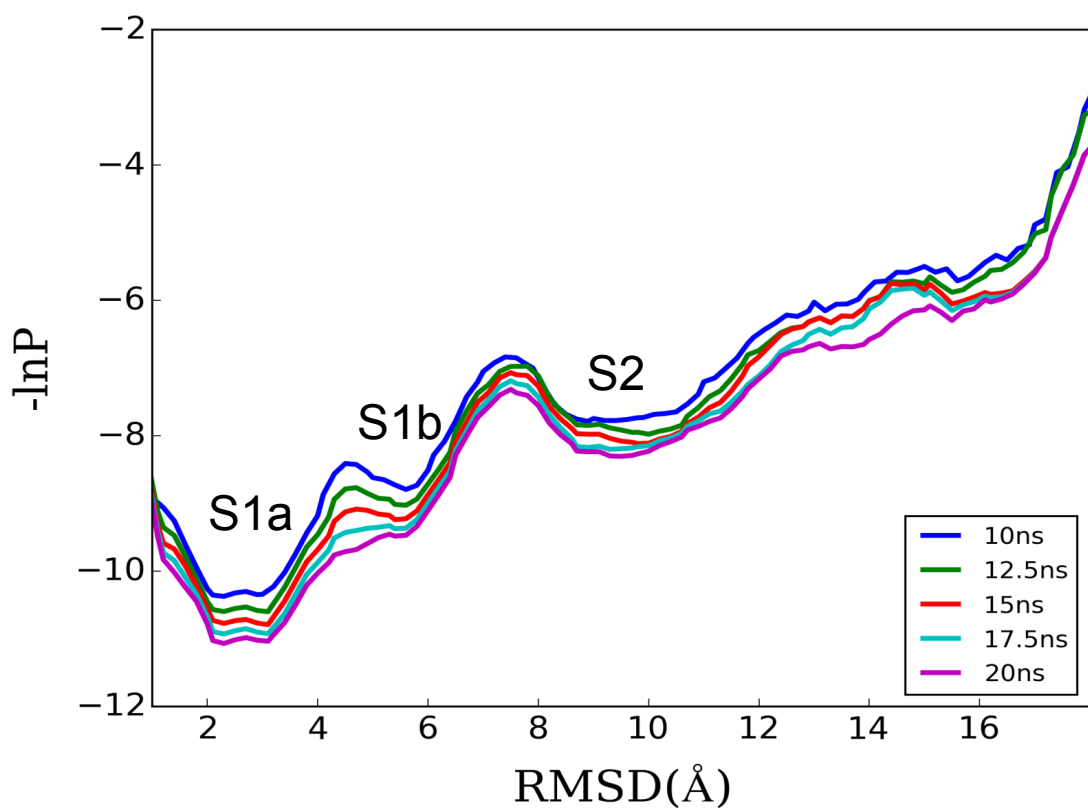

Supplement: S2 Fig — Each population density profile was obtained as that in main Fig 2, projected along the PPi RMSD coordinate. The results were obtained from 100 trajectories, started from 5 to 20 ns (upper panel) and 10 to 20 ns (lower panel) for each individual trajectory. The convergence is improved from 10 to 20 ns around the S1a, S1b, and S2 metastable states. (PDF) [file pcbi.1004624.s003.pdf]

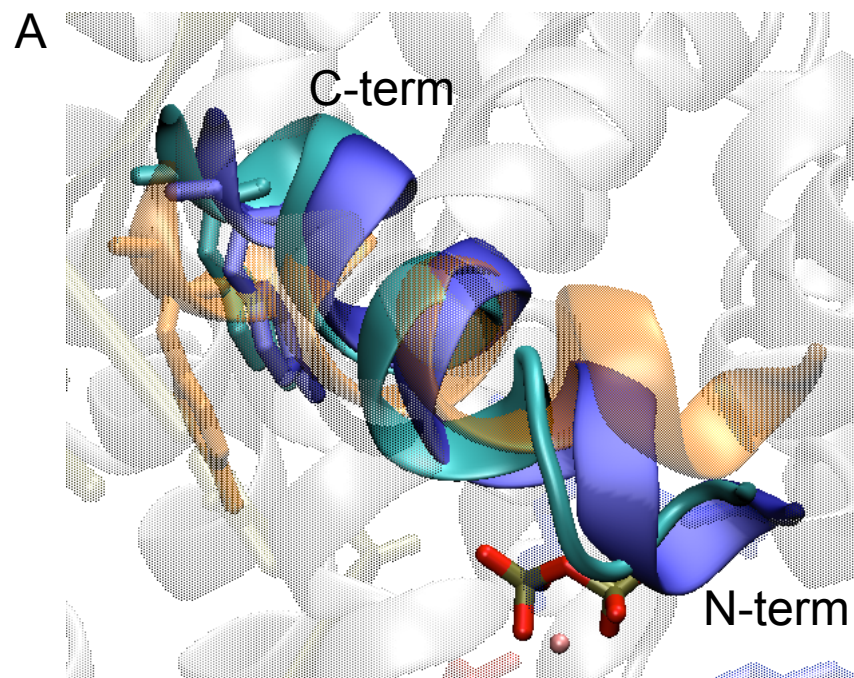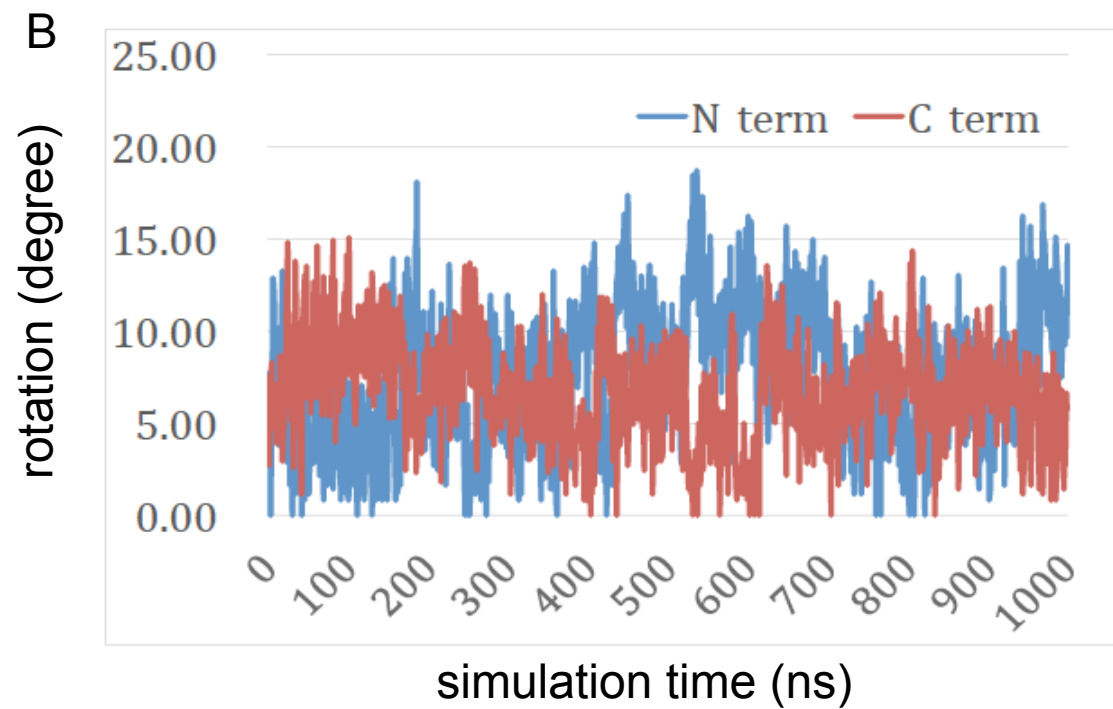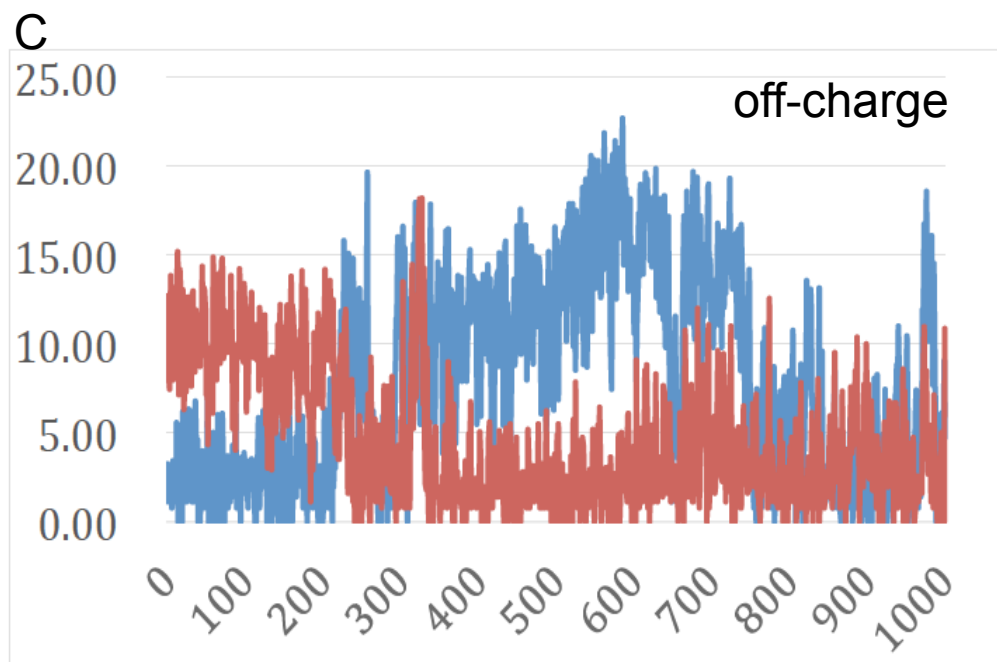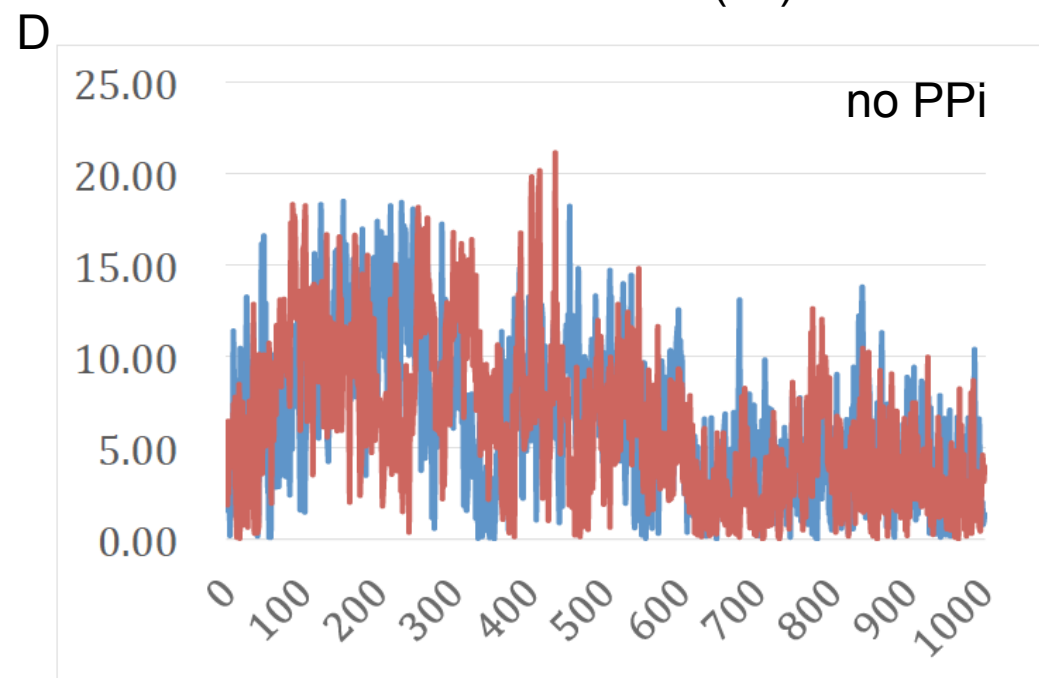

Supplement: S3 Fig — (A) The molecular view of the O-helix in different configurations are shown: An initial closed configuration (cyan), a random configuration picked from the unperturbed MD simulation (blue), and an open configuration (orange). The PPi-MgB group is shown in licorice. The rotation angles measured from the N-term (blue) and the C-term (red) were calculated from the long MD simulations, for the unperturbed product complex (B), the off-charge complex (C), and the product complex with PPi removed (D). The way calculating the O-helix rotation is illustrated in S1 text. (PDF) [file pcbi.1004624.s004.pdf]

**A**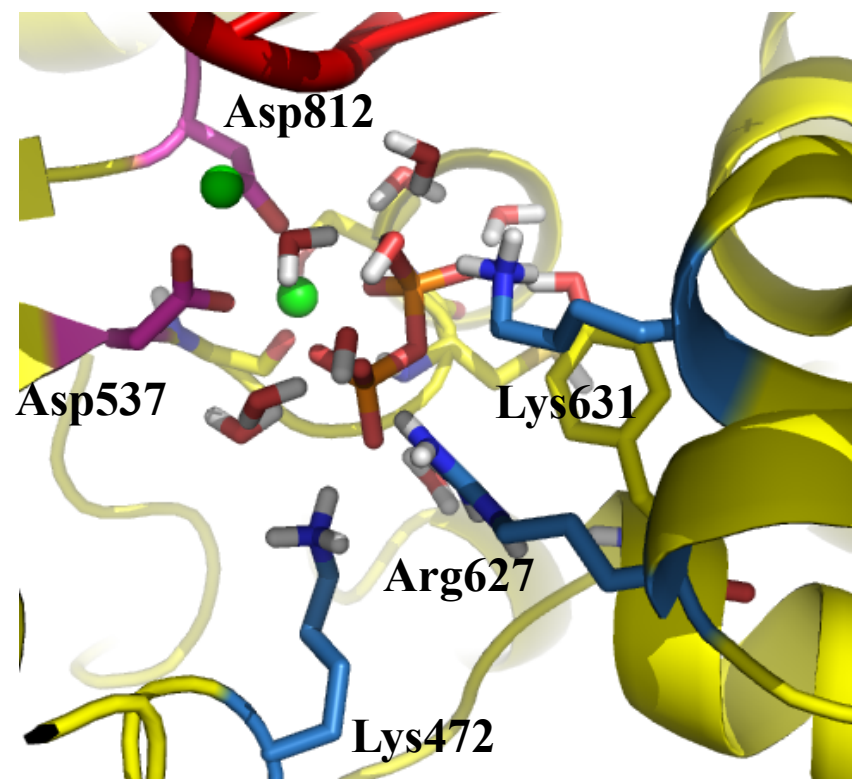**B**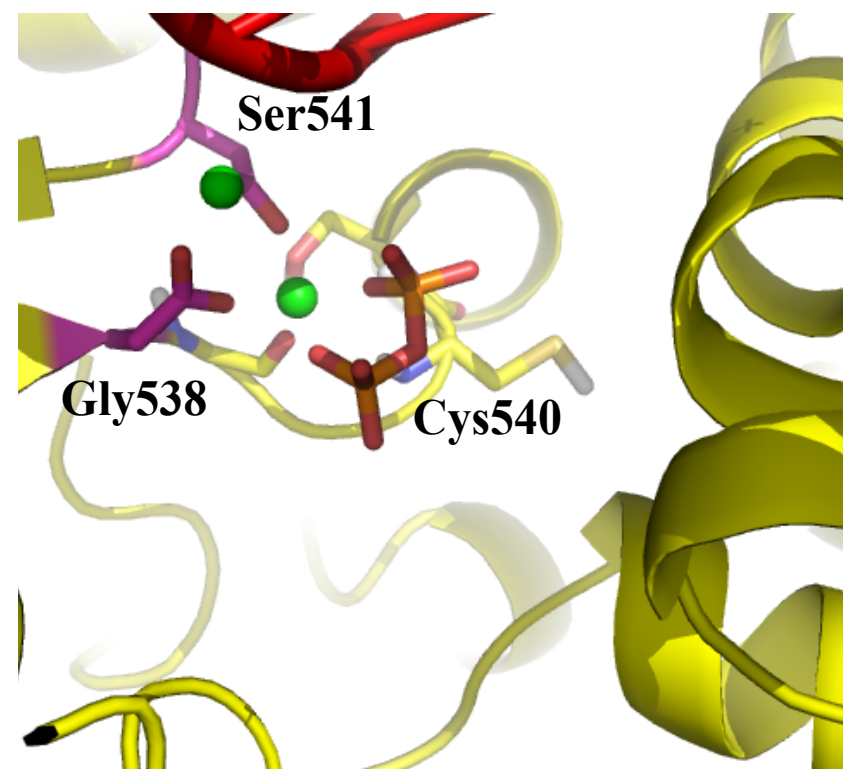**C**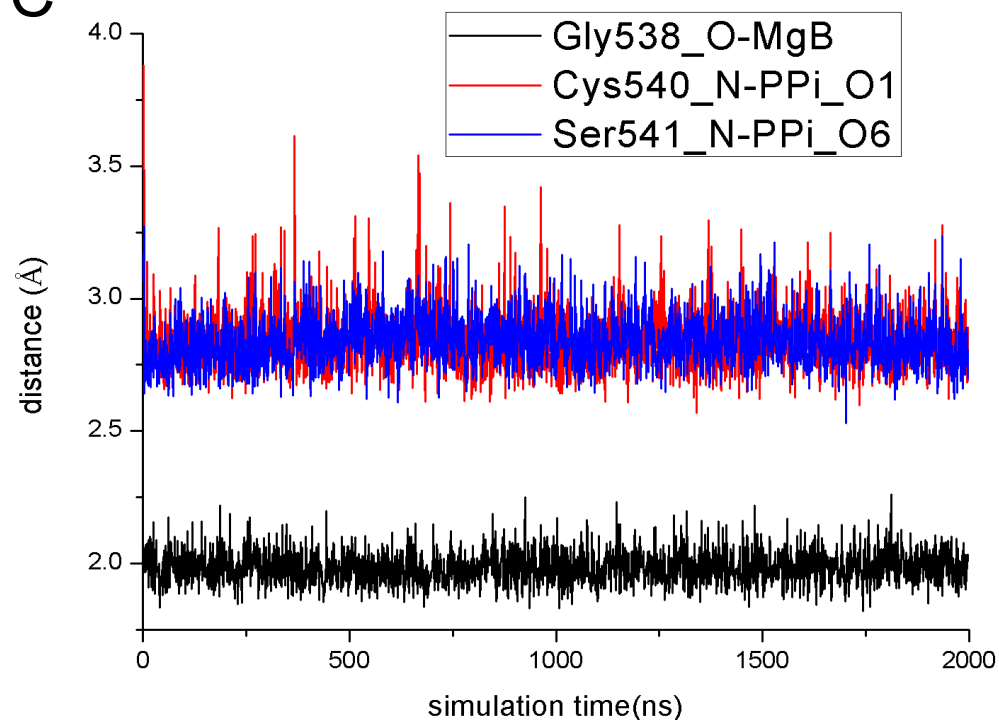**D**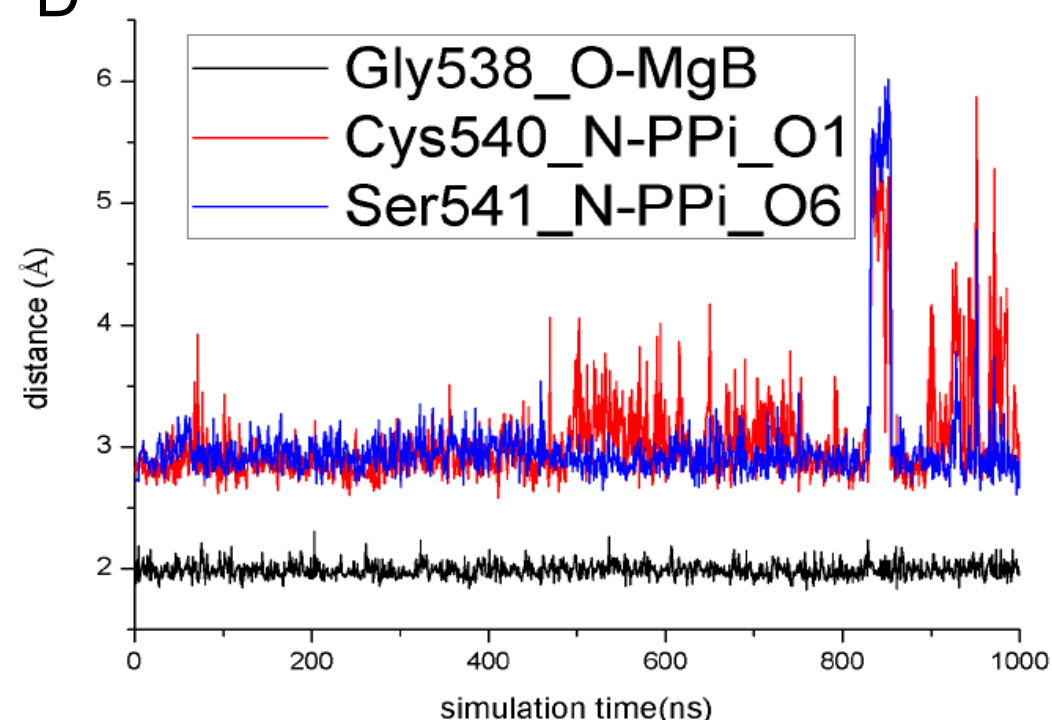

Supplement: S4 Fig — (A) In addition to the residues Lys472, Arg627, Lys631, Asp537 and Asp812, several water molecules can enter into the active site and interact with the (PPi-MgB)2- group. (B) Moreover, the backbone oxygen atom of residue Gly538 can coordinate with the MgB atom, and residues Cys540 and Ser541 can form hydrogen bonds with PPi through the N-H group of the amide bond. (C & D) The distances from the non-charged local residues Gly538, Cys540 and Ser541 to (PPi-MgB)2-, in both the unperturbed complex (C) and the off-charge complex (D). (PDF) [file pcbi.1004624.s005.pdf]

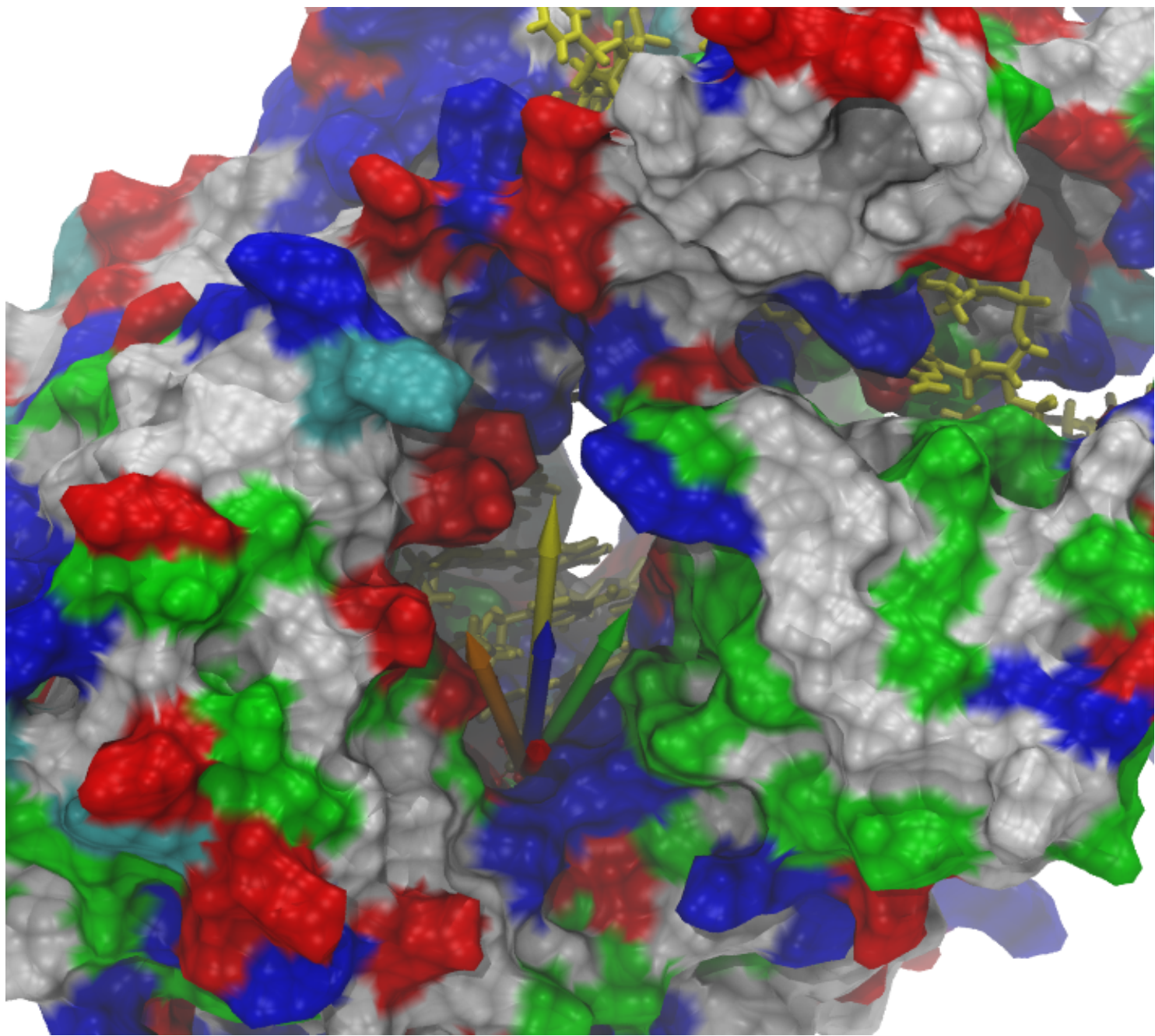

Supplement: S5 Fig — The pulling started from the initial position of PPi, and was made towards the COM of the Cα atoms of five different combinations of residues (see text), along direction 1 (red arrow), direction 2 (orange), direction 3 (yellow), direction 4 (green), and direction 5 (blue). The polymerase is shown in surface representation and colored according to the charge of residues (blue: positive; red: negative; green, polar; white: nonpolar). (PDF) [file pcbi.1004624.s006.pdf]

**A**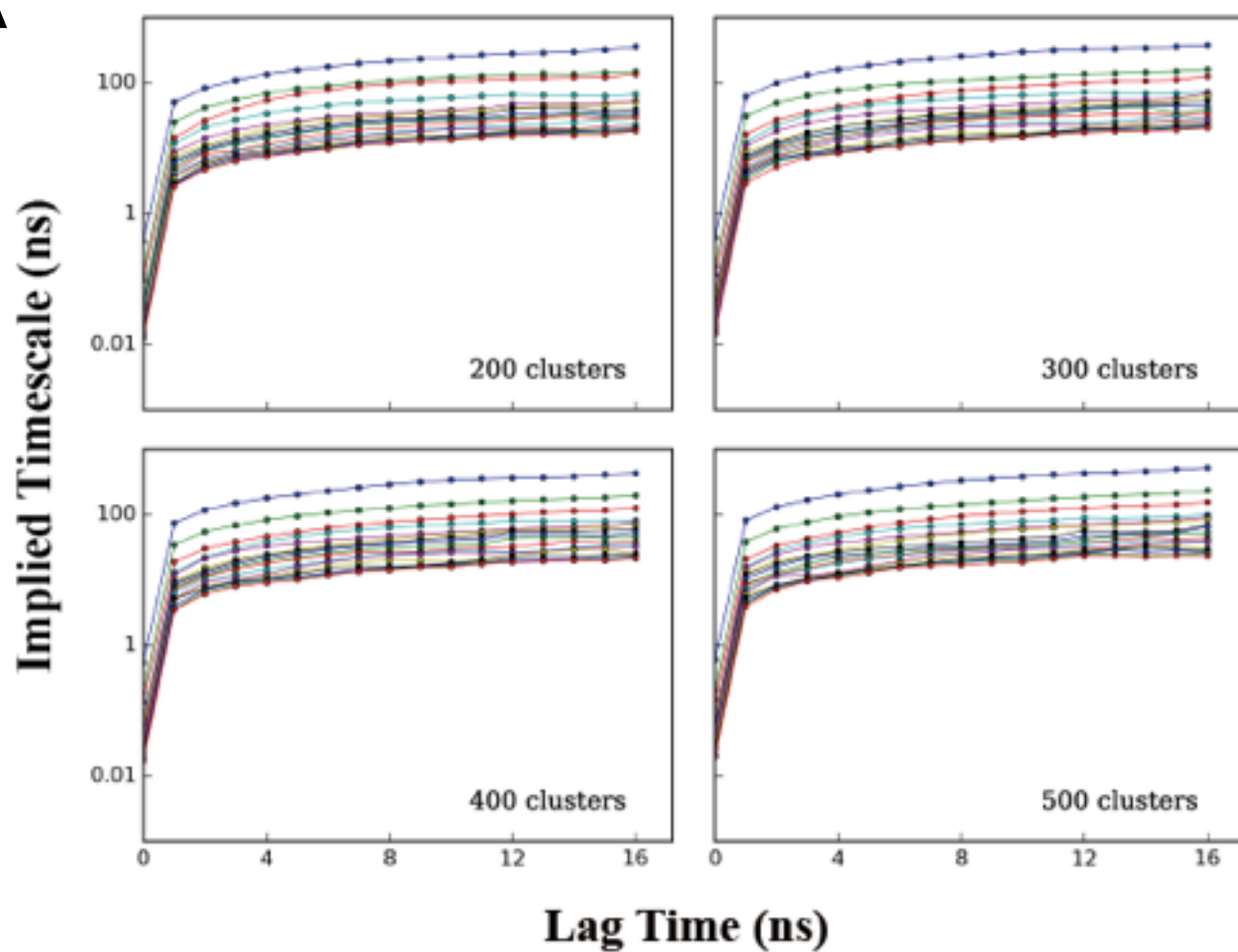**B**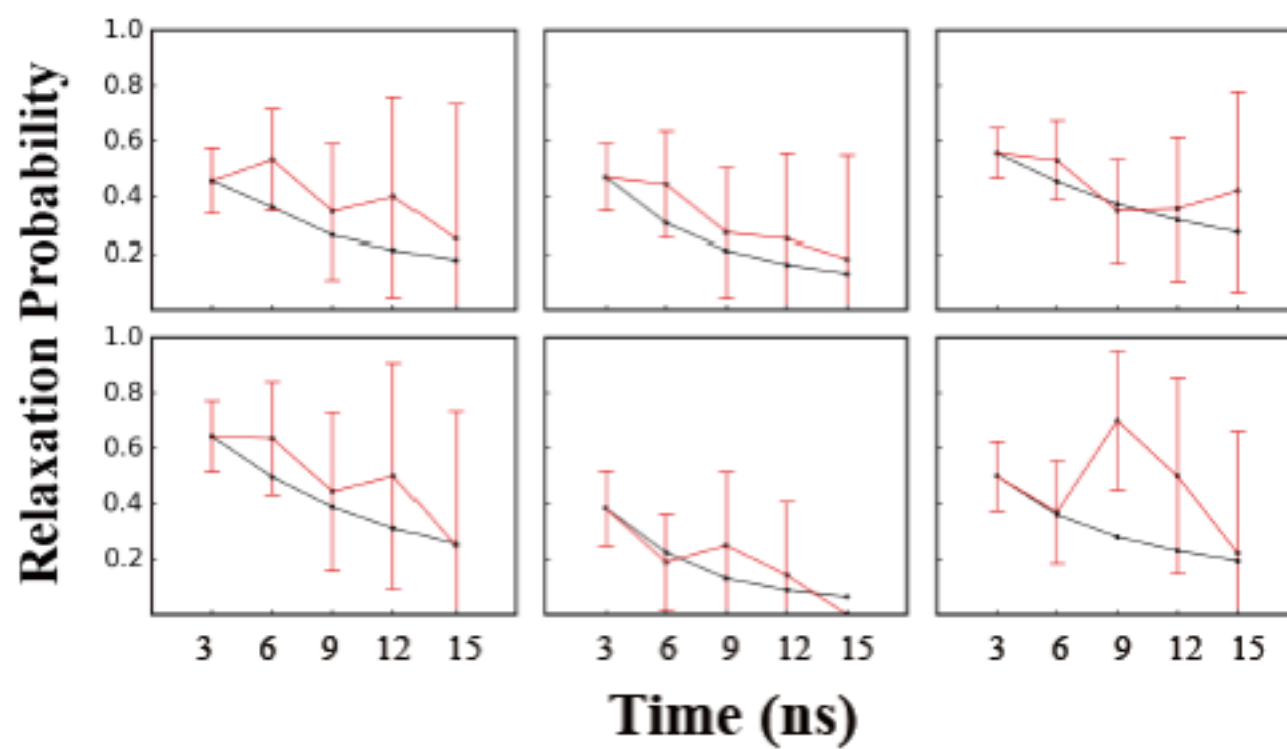

Supplement: S6 Fig — (A) The implied timescales as the function of different lag time for the 200, 300, 400 and 500 microstate models, respectively. (B) For six most populated microstates, we predicted the self-transition probability for each state after a lag time of 3ns based on our 200-state MSM (blue line). The results were compared with the corresponding values counted directly from the MD simulations (red line). (PDF) [file pcbi.1004624.s007.pdf]

log Counts

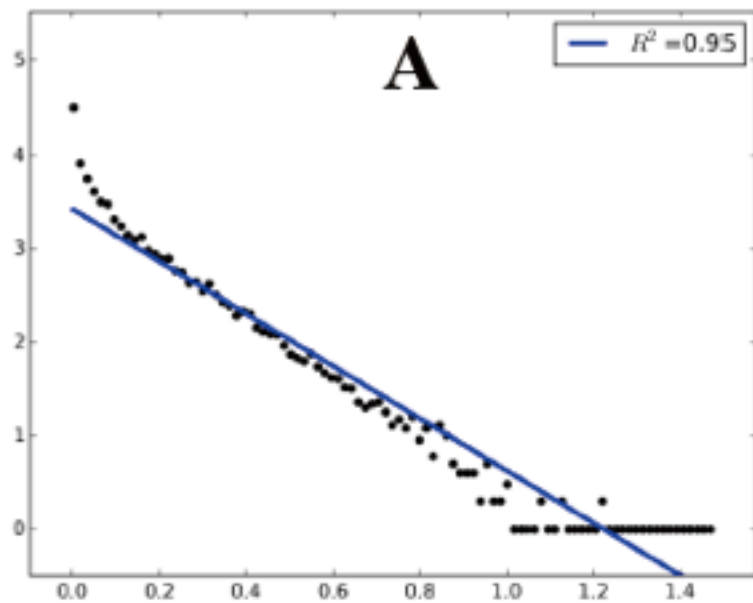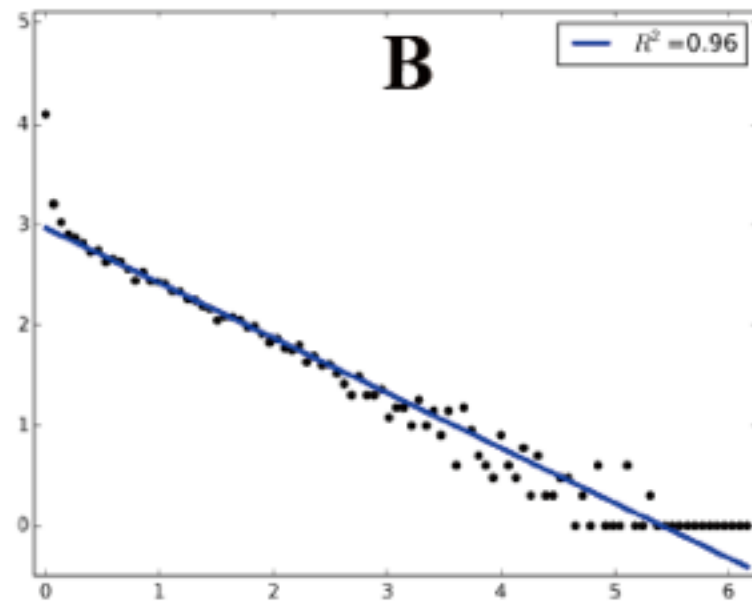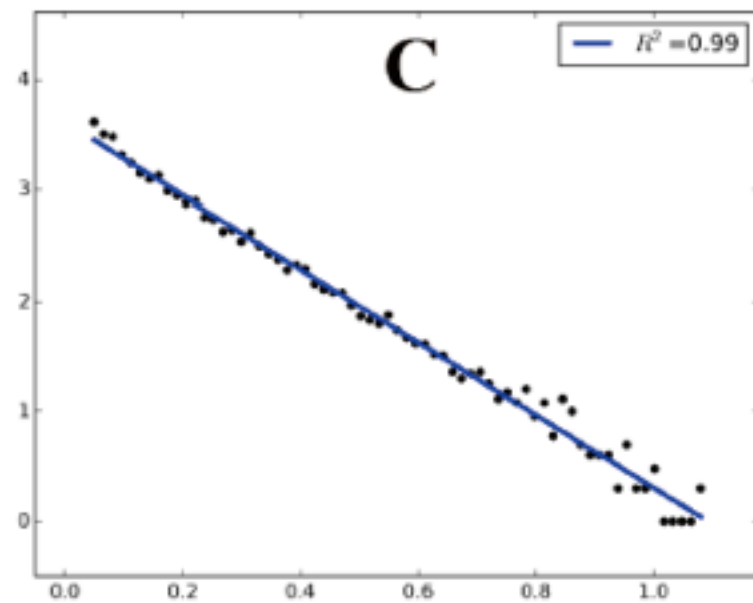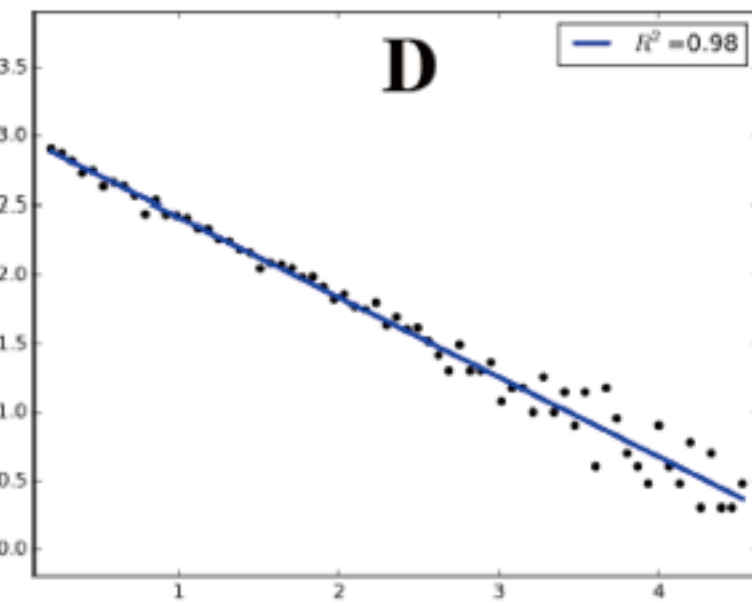

MFPT ( $\mu\text{s}$ )

Supplement: S7 Fig — Original MFPT counts (logarithm) in the MC trajectory for the S1a to S1b (A) and S1b to S2 (B) transitions. Then, for each of the above two transitions, better linear regression was performed by truncating several MFPT values that correspond to either fastest or slowest dynamics (C and D, respectively). (PDF) [file pcbi.1004624.s008.pdf]

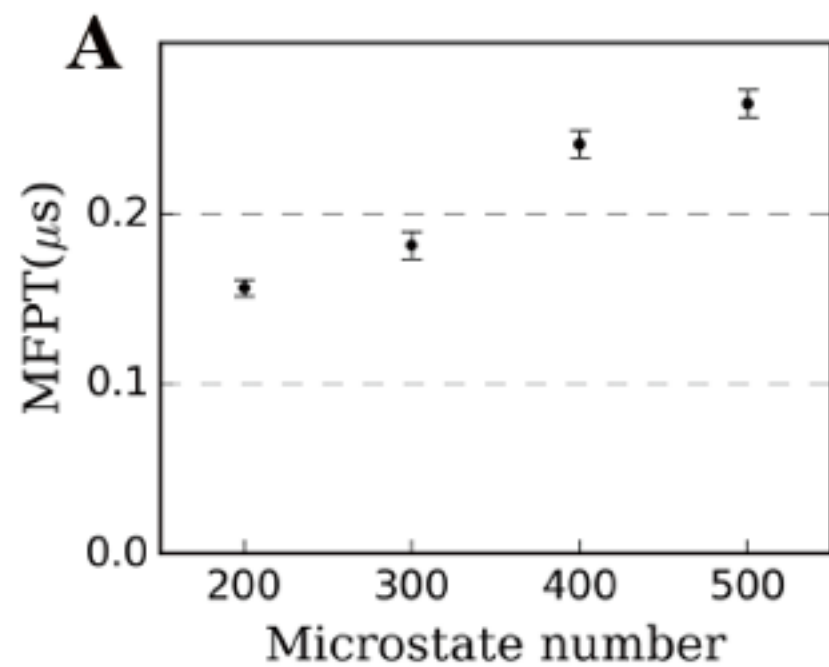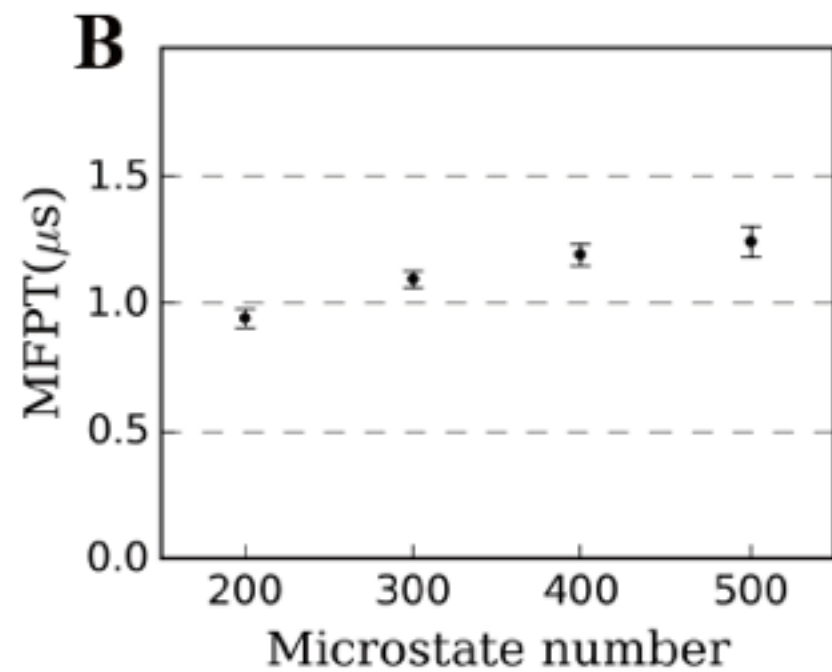

Supplement: S8 Fig — For each model, the average and standard error of the MFPT were calculated by generating ten parallel 10 ms MC long trajectories that were built based on the corresponding transition probability matrix of MSM (see main Methods section for more details). (PDF) [file pcbi.1004624.s009.pdf]

**A**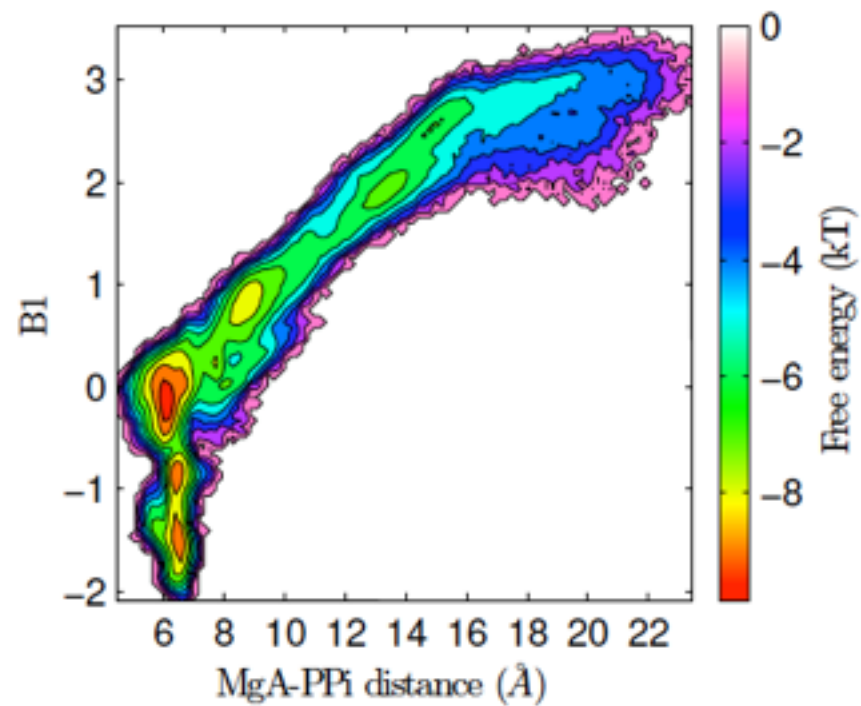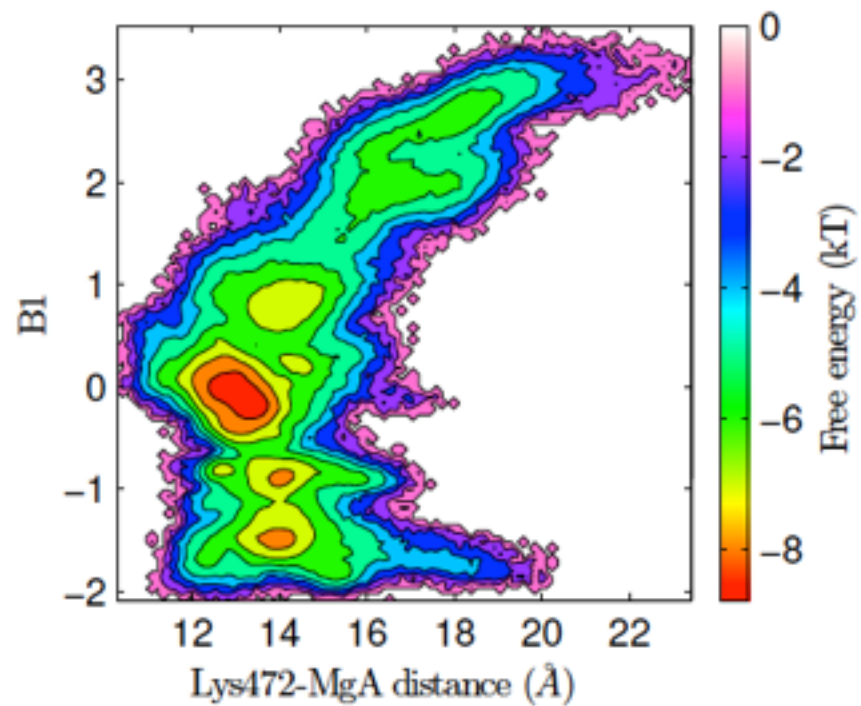**B**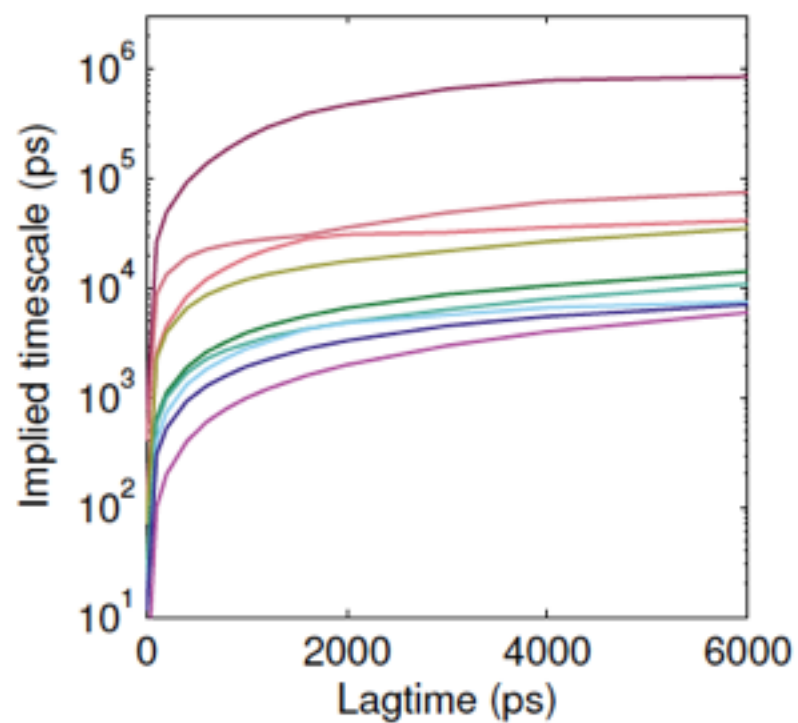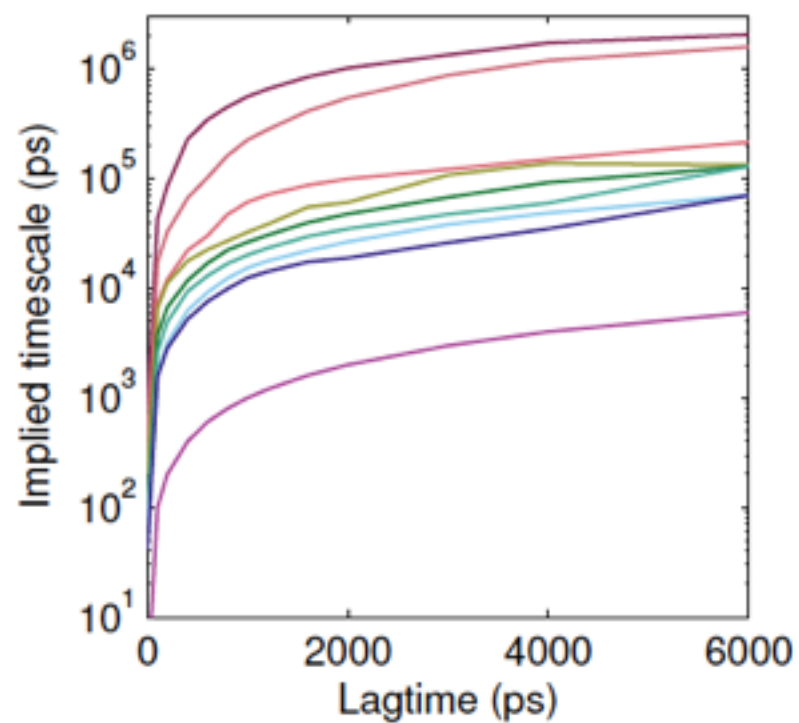

Supplement: S9 Fig — (A) The 2D population density maps (- lnP) along the slowest mode (B1) & the MgA-PPi distance (left), and along B1 & the Lys472-MgA distance (right). B1 appears to describe the PPi release equivalently well with the other two coordinates. Five metastable states reveal, with three of them within S1a, while the other two corresponding to S1b and S2 respectively from the MSM (see S1A Fig). (B) The implied time scales calculated according to the slowest mode (B1; left) and according to the five slowest modes (B1 to 5; right). (PDF) [file pcbi.1004624.s010.pdf]
